# The evolutionary origins of streptophyte multicellularity

**DOI:** 10.1111/nph.71415

**Published:** 2026-07-09

**Authors:** Tatyana Darienko, Cäcilia F. Kunz, Elisa S. Goldbecker, Erika Endriulaitytė, Maaike J. Bierenbroodspot, Maike Lorenz, Iker Irisarri, Thomas Pröschold, Katharina Bürstenbinder, Henrik Buschmann, Jan de Vries

**Affiliations:** ^1^ Department of Applied Bioinformatics Institute for Microbiology and Genetics, University of Göttingen Julia‐Lermontowa‐Weg 3 37077 Göttingen Germany; ^2^ Department of Experimental Phycology and SAG Culture Collection of Algae Albrecht‐von‐Haller‐Institute for Plant Sciences, University of Göttingen Nikolausberger Weg 18 37073 Göttingen Germany; ^3^ Department of Biodiversity and Evolutionary Biology Museo Nacional de Ciencias Naturales (MNCN‐CSIC) José Gutiérrez Abascal 2 28006 Madrid Spain; ^4^ Research Department for Limnology University of Innsbruck Mondseestr. 9 5310 Mondsee Austria; ^5^ Plant Cell Biology University of Marburg Karl‐von‐Frisch‐Straße 8 35043 Marburg Germany; ^6^ Plant Physiology, Institute of Biology Martin‐Luther‐University Halle‐Wittenberg Weinbergweg 10 06120 Halle (Saale) Germany; ^7^ Faculty of Applied Computer Sciences and Biosciences, Section Biotechnology and Chemistry, Molecular Biotechnology University of Applied Sciences Mittweida Technikumplatz 17 09648 Mittweida Germany; ^8^ University of Göttingen, Campus Institute Data Science (CIDAS) Julia‐Lermontowa‐Weg 3 37077 Göttingen Germany; ^9^ Department of Applied Bioinformatics Göttingen Center for Molecular Biosciences (GZMB), University of Göttingen Julia‐Lermontowa‐Weg 3 37077 Göttingen Germany

**Keywords:** multicellularity, plant cell biology, plant evolution, plant terresrialization, Streptophyte algae

## Abstract

Over their more than 1 billion years of evolutionary history, streptophytes have repeatedly transitioned between unicellular, simple multicellular and complex multicellular forms. Rather than a linear trajectory toward increasing complexity culminating in land plants, streptophyte evolution is now understood as highly dynamic. Phylogenomic analyses reveal an early origin of multicellularity followed by lineage‐specific losses, reductions and secondary gains of complexity. Filamentous and parenchymatous body plans in Charophyceae and Coleochaetophyceae are derived rather than direct precursors of land plant multicellularity, while zygnematophyte algae – the sister group to embryophytes – exhibit dramatic reductions to unicellular or filamentous forms despite retaining genetic toolkits linked to multicellular traits. We argue that streptophyte multicellular evolution is best explained by regulatory changes at the cellular level rather than gene gain or loss alone. Variation in cell division patterns, polarity establishment, cell wall sensing and intercellular connectivity plays a central role in shaping body plan diversity. We further propose that rewiring protein–protein interaction networks, mediated by intrinsically disordered regions and short linear motifs, represents a key mechanism driving repeated evolutionary innovation. Integrating comparative genomics, ancestral state reconstruction and functional cell biology will clarify how conserved molecular toolkits generated streptophyte diversity and ultimately facilitated the emergence of land plants.

## Graphical abstract

The phylogeny of streptophytes and the deep origin of branching filamentous growth.

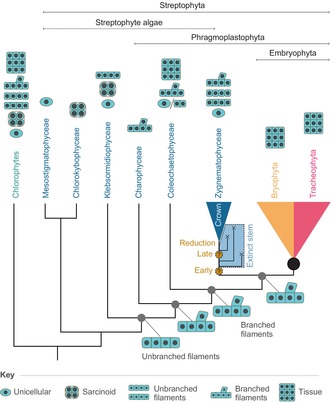

## Zusammenfassung

Im Verlauf ihrer mehr als eine Milliarde Jahre umfassenden Evolutionsgeschichte haben Streptophyten wiederholt Übergänge zwischen einzelligen, einfach vielzelligen und komplex vielzelligen Organisationsformen vollzogen. Anstatt einer linearen Entwicklung hin zu zunehmender Komplexität, die schließlich in den Landpflanzen gipfelt, wird die Evolution der Streptophyten heute als hochdynamischer Prozess verstanden. Phylogenomische Analysen weisen auf einen frühen Ursprung der Vielzelligkeit hin, auf den stammlinienabhängige Verluste, Reduktionen und sekundäre Zugewinne an Komplexität folgten. Die filamentösen und parenchymatischen Baupläne der Charophyceae und Coleochaetophyceae sind demnach abgeleitete Formen und keine direkten Vorläufer der Vielzelligkeit der Landpflanzen. Demgegenüber zeigen die Zygnematophyceae – die Schwestergruppe der Embryophyten – eine ausgeprägte Reduktion zu einzelligen oder filamentösen Formen, obwohl sie weiterhin genetische Ausstattung besitzen, die mit vielzelligen Merkmalen in Zusammenhang steht. Wir vertreten die Auffassung, dass die Evolution der Vielzelligkeit bei den Streptophyten am besten durch Veränderungen der zellulären Regulation erklärt werden kann und nicht allein durch den Gewinn oder Verlust von Genen. Unterschiede in den Mustern der Zellteilung, der Etablierung von Zellpolarität, der Wahrnehmung der Zellwand sowie der interzellulären Konnektivität spielen eine zentrale Rolle bei der Ausbildung der Vielfalt von Körperbauplänen. Darüber hinaus schlagen wir vor, dass die Umgestaltung von Protein–Protein‐Interaktionsnetzwerken, vermittelt durch intrinsisch ungeordnete Regionen und kurze lineare Motive, einen entscheidenden Mechanismus für wiederholte evolutionäre Innovationen darstellt. Die Verknüpfung von vergleichender Genomik, Rekonstruktionen von anzestralen Zuständen und funktioneller Zellbiologie wird dazu beitragen zu klären, wie konservierte molekulare Werkzeugsätze die Diversität der Streptophyten hervorbrachten und letztlich die Entstehung der Landpflanzen ermöglichten.

**Table:** 

	Contents	
	Summary	2365
I.	Introduction	2366
II.	A deep multicellular backbone of streptophytes	2368
III.	A preamble on trait complexity and regulation	2370
IV.	Biophysical feedback mechanisms and sensing of the status of the cell	2372
V.	Polarity: a molecular nexus in complexity of streptophyte cell biology and physiology	2372
VI.	Dividing the cell: a concerted cell biological process	2374
VII.	Unique events and unicellular complexity shape the evolution of the Zygnematophyceae	2375
VIII.	Connectivity, not complexity?	2376
IX.	Gain vs tinkering	2377
X.	Conclusion	2378
	Acknowledgements	2378
	References	2379

*This happened, for instance, during one of the main events of cellular evolution: namely, the passage from unicellular to multicellular forms. This was a particularly important transition because it carried an enormous potential for a specialization of the parts. Such a transition, which probably occurred several times, did not require the creation of new chemical species, for there are no major differences between molecular types of uni‐ and multicellular organisms. It was mainly a reorganization of what already existed*. (Jacob, 1977)

## I. Introduction

**Fig. 1:**
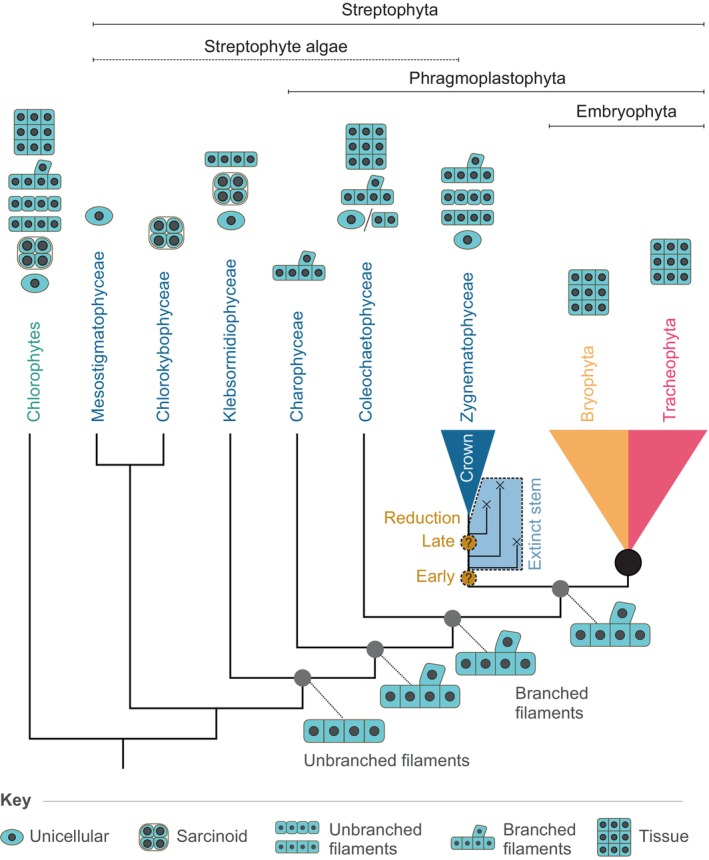
Phylogeny of streptophytes and the deep origin of branching filamentous growth. A simplified cladogram based on our current understanding of the phylogenetic relationships within streptophytes (see main text). Belonging to the green lineage, the monophylum Streptophyta likely emerged far more than 1 billion years ago (Bowles *et al*., 2024b) and diversified over the course of evolution into six streptophyte algal classes that form a monophylum with the land plants (Embryophyta). Three algal classes and the monophyletic land plants form the monophylum Phragmoplastophyta. In this article we argue that the last common ancestor (LCA) of Klebsormidiophyceae and Phragmoplastophyta likely had unbranched filaments, whereas all the LCAs of Phragmoplastophyta had branched filaments.

**Fig. 2:**
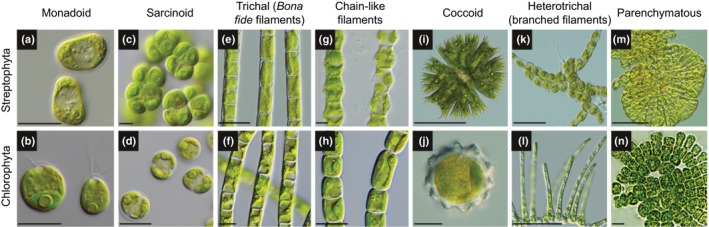
Different growth types re‐emerged during the evolution of streptophytes and chlorophytes. Exemplary micrographs of streptophyte and chlorophyte algae with different body plan organizations. The same types of (analogous) body plans have independently emerged in these major groups of Chloroplastida. (a) *Mesostigma viride* SAG 50‐1, (b) *Edaphochlamys debaryana* SAG 4.72, (c) *Chlorokybus cerffii* SAG 34.98, (d) *Chlorosarcinopsis sempervirens* SAG 214‐1, (e) *Klebsormidium flaccidum* SAG 335‐7, (f) *Geminella* sp. SAG 25.94, (g) *Bambusina brebissonii* SAG 20.82, (h) *Geminella minor* SAG 22.88, (i) *Micrasterias fimbriata* SAG 162.80, (j) *Trochisciopsis tetraspora* SAG 19.95, (k) *Coleochaete divergens* SAG 32.97, (l) *Stigeoclonium aestivale* SAG 410‐1, (m) *Coleochaete scutata* SAG 2665, (n) *Chaetopeltis orbicularis* ACOI‐1728. All micrographs (a–m) were taken by T. Darienko, except for the one of *Chaetopeltis* (n), which was photographed by M. F. Santos, Coimbra Collection of Algae (ACOI), University of Coimbra, Portugal (used here with permission). Bars, 10 μm (all pictures except: (i) 100 μm; (k, l) 50 μm; (m) 200 μm).

More than a billion years ago, the green lineage split into the clades Chlorophyta and Streptophyta (Morris *et al*., 2018; Donoghue *et al*., 2021; Strassert *et al*., 2021; Bierenbroodspot *et al*., 2024a). The Streptophyta have massively radiated into six major classes of streptophyte algae and, its most famous lineage, the Embryophyta or land plants (One Thousand Plant Transcriptomes, 2019; Bierenbroodspot *et al*., 2024b; Fig. 1). The origin of embryophytes is a classic among the major questions in evolutionary biology (Graham *et al*., 2000; de Vries & Archibald, 2018; Bowman, 2022), representing a singularity in the diversification of species, genes and traits (Bowles *et al*., 2020; Donoghue *et al*., 2021; Harris *et al*., 2022; Clark *et al*., 2023). While embryophytes are the only photosynthetic eukaryotes with macroscopic multicellular bodies that have fully conquered the terrestrial habitat on a global scale, they are neither the only multicellular streptophytes nor the only terrestrial ones (Lewis & McCourt, 2004; Fürst‐Jansen *et al*., 2020; McCourt *et al*., 2023): within the green lineage the occurrence of algae that have diverse multicellular bodies and/or live in terrestrial habitats is not limited to streptophytes. Also, throughout the evolution of chlorophytes, diverse terrestrial adaptations (Karsten & Holzinger, 2014) and complex bodies emerged (Fig. 2; Table 1; Umen, 2014; Umen & Herron, 2021). In the following, we will unpack this with a focus on the streptophyte algae.

**Table 1:** Iconic species and strains illustrating different stages of body plan organization.

	Unicellular		Sarcinoid		Unbranched filaments		Branched filaments		Tissue	
	Species	Strain #	Species	Strain #	Species	Strain #	Species	Strain #	Species	Strain #
Mesostigmatophyceae	*Mesostigma viride*	SAG 50–1	–		–		–		–	
Chlorokybophyceae	–	–	*Chlorokybus cerffii*	SAG 34.98	–		–		–	
			*Chlorokybus riethii*	SAG 48.80						
Klebsormidiophyceae	*Interfilum paradoxum*	SAG 338–1	*Interfilum massjukiae*	SAG 2102	*Klebsormidium flaccidum*	SAG 2307	–		–	
			*Streptosarcina arenaria*	SAG 2560, SAG 2562	*Klebsormidium nitens*	SAG 335–1a				
			*Streptosarcina costaricana*	SAG 36.98, SAG 2653	*Hormidiella parvula*	SAG 2558				
					*Entransia fimbriata*	UTEX 2353, UTEX 2793				
Coleochaetophyceae	–		–		–		*Chaetosphaeridium globosum*	SAG 26.98	*Coleochaete scutata*	SAG 110.80
							*Coleochaete divergens*	SAG 36.97		
Charophyceae	–		–		–		*Chara braunii*	S276, S277	–	
Zygnematophyceae	*Mesotaenium endlicherianum*	SAG 12.97	–		*Spirogyra pratensis*	MZCH‐10213	–		–	
	*Closterium psl*.	NIES‐68			*Zygnema circumcarinatum*	SAG 698‐1b				
	*Penium margaritaceum*	SAG 2640			*Mougeotiopsis calospora*	MZCH‐580				

The streptophyte algae consist of six independent lineages from unicellular flagellate (*Mesostigma*) to complex thalli (*Chara*). Each lineage is considered a class. The two classes Mesostigmatophyceae and Chlorokybophyceae form the sister group to all other streptophytes (Marin & Melkonian, 1999; Irisarri *et al*., 2021). *Mesostigma* is a lineage with only two species (Fig. 2a) and the only monadoid (a single‐celled flagellate), resembling chlorophytes like *Edaphochlamys* (Fig. 2b). The flagellated *Mesostigma* cells have the characteristic streptophytic unilateral basal body orientation, which is rooted with the multilayered structure (MLS; Manton & Ettl, 1965). This flagellar apparatus also occurs in the zoospores of the Chlorokybophyceae, a class that contains five morphologically cryptic species of sarcinoid organization (Rogers *et al*., 1980; Lokhorst *et al*., 1988; Irisarri *et al*., 2021; Fig. 2c), which occurs in chlorophytes for example in *Chlorosarcinopsis* (Fig. 2d). Sarcinoid cell packages are held together by a common mucilage layer and could be therefore considered a multicellular assemblage.

Klebsormidiophyceae contains three types of cellular organization: unicells to short filaments in *Interfilum*, cell packages in *Interfilum* and *Streptosarcina*, and long unbranched filaments in *Klebsormidium* (Fig. 2e), *Hormidiella* and *Entransia*; similar filaments occur in the chlorophyte *Geminella* (Fig. 2f). The cells in the sarcinoid taxa are arranged similarly to those in *Chlorokybus*. The multicellularity of filamentous species is brought about by different cell wall layers: Whereas the inner layers of the cell wall take part in cell division, the outer layers cover both daughter cells without taking part in division (Floyd *et al*., 1972; Lokhorst & Star, 1985). As a result, the cells stay together, but they are not connected by plasma bridges (plasmodesmata). In this class, the first indication of cell differentiation occurs by formation of stalks (rhizoid‐like structure) for settlement on surfaces. Cells at the end of the filament appear rounded, as in *Hormidiella* and *Entransia* (Bierenbroodspot *et al*., 2024a, 2024b) – the rounded appearance likely results from a lack of physical counterforces that keep the weaker cross walls in shape (for a characterization of these cross walls, see Herburger & Holzinger, 2015). Despite all cells being able to divide, cells that face the light show higher rates of division (Lokhorst, 1996). Altogether, this warrants attention. It is from these pieces – tissue context combined with polarity, for example directed by light – that might have provided building blocks for what became apical growth – more on this in Sections IV and V.

The most complex streptophyte algae bodies occur in the Charophyceae, which form macroscopic thalli. The erect thalli exhibit various stages of cell differentiation, including rhizoids, nodal and internodal cells, stipulae and others. They also form unique sexual organs, such as archegonia and antheridial stands. All cells in the vegetative thallus are connected by plasmodesmata. Historically, Charophyceae were considered to be the closest relatives to embryophytes because of their complexity (Pringsheim, 1862), yet this was debunked by recent phylogenetic analyses – more on this below and Section II.

The second closest algal relatives to land plants are the Coleochaetophyceae, a class represented by the two genera *Chaetosphaeridium* and *Coleochaete* (Bierenbroodspot *et al*., 2025). Both genera form branched filaments that frequently exhibit specialized cells that bear long hairs, readily occurring under nutrient (phosphate) depletion (Printz, 1964). These hairs probably serve to increase the surface of the cell bodies. *Coleochaete scutata* forms discoid parenchymatous thalli. All taxa of this class have plasmodesmata.

The sister group of the Embryophyta are the Zygnematophyceae, a class comprising unicellular and unbranched filaments, which are subdivided into five orders (Hess *et al*., 2022): Desmidiales, Serritaeniales, Zygnematales, Spirogyrales and Spirogloeales. Zygnematophyceae can live in diverse and even extreme habitats (Pichrtová *et al*., 2018; Rippin *et al*., 2019; Bowles *et al*., 2024a; Kunz *et al*., 2024b) – including the zygnematophyceaen algae growing on ice (Remias *et al*., 2012a; Remias & Procházková, 2023; Procházková *et al*., 2025). Body plans of coccoid and trichal organization can be found throughout the different orders. The largest diversity of species belongs to the Desmidiales, which mostly contain coccoids of intricate form (cells consisting of the (often) ornamented semi‐cells). The filamentous taxa in this order are formed by what are considered loose connections, resulting in chains of cells. True filaments mainly exist in the orders Zygnematales and Spirogyrales, whereas Serritaeniales and Spirogloeales are dominated by unicellular taxa. The only filamentous species of the Serritaeniales is *Mougeotiopsis calospora* (Palla, 1894; Hess *et al*., 2022). In Zygnematophyceae, filaments are either trichal or chain‐like (Fig. 2g); examples of chlorophyte chain‐like filaments occur in certain *Geminella* (Fig. 2h). The formation of plasmodesmata in the Zygnematophyceae has not been reported. In contrast to the other streptophyte classes, no flagellated cells have been reported in the zygnematophytes. Unique in this class is also the type of cell division in the Desmidiales (more on this below) and the sexual reproduction by conjugation (van den Hoek *et al*., 1995). Indeed, despite being unicells, the desmids are extremely complex cells with an ornamentation that suggests intricate cell biology (Fig. 2i); similar ornamental patterns are found in the chlorophyte *Trochisciopsis* (Fig. 2j).

Before the dawn of molecular phylogenetics, the trajectory of streptophyte evolution was predominantly conceptualized as a progressive gain of morphological complexity (Pringsheim, 1862). Yet, we now know that complex streptophyte algal macrophytes like *Chara* represent secondary gains of morphogenetics, as evident from their independently expanded gene repertoires (Nishiyama *et al*., 2018) and their phylogenetic position (Wodniok *et al*., 2011; Wickett *et al*., 2014). In fact, current estimates recover an ancient origin of multicellularity of streptophytes (Bierenbroodspot *et al*., 2024a; Bowles *et al*., 2024b), underpinned by an ancient toolkit (Mulvey & Dolan, 2023). Further, the Coleochaetophyceae, renowned for their morphological complexity, are mostly branching filaments (Fig. 2k; branching is also found in chlorophytes, see Fig. 2l). Coleochaetophyceae have gained their signature discoidal growth (Fig. 2m) only recently – an innovation within the clade (Bierenbroodspot *et al*., 2025) and something mirrored in the chlorophyte *Chaetopeltis* (Fig. 2n).

**Fig. 3:**
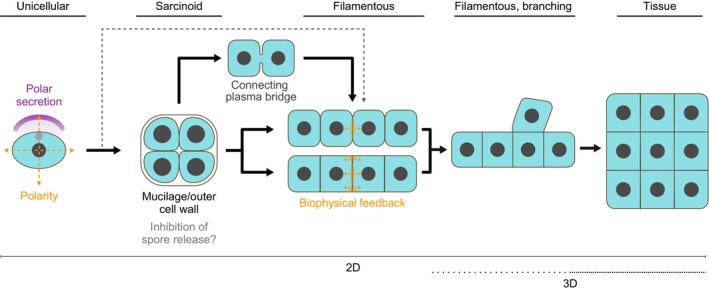
Trajectories in the evolution of multicellularity and complexity. A conceptual figure that illustrates that the evolution of multicellularity and complex body plans seems to follow similar tracks. The basis is polarity, cell–cell adherence, connection and communication. Biophysical feedback between adjacent cells – and parts of the cell–cell interface are key. This biophysical feedback is likely intertwined with cell wall integrity sensing and distinguishing between inside and outside. The latter is key for protection and even streptophyte unicells show polar responses (such as secretion of sunscreens) toward challenges from the outside (likely extending to other factors, such as predators and mechanical stimuli).

Overall, a picture comes into focus that describes a deep origin of multicellularity in streptophytes, with a dynamic evolution of its actualization across lineages that also independently occurred in chlorophytes (Fig. 3); this actualization describes the genetic event that converted latent evolutionary potential into a concerted expression that underpins the complex trait of multicellularity (for a sleek example of how this can manifest, see Blount *et al*., 2012). In this article, we will ponder the implications for our understanding of streptophyte evolution. We will further outline a path to move this research forward, disentangling the complexities underpinning the evolution of streptophyte body plans.

## II. A deep multicellular backbone of streptophytes

Tracing trait evolution requires ancestral character state reconstruction. When working with extant species from which we garner molecular data, this means mapping character states of said extant organisms onto the leaves of phylogenetic trees, walking backwards to ancestral nodes and inferring their character states using probabilistic methods. This endeavor requires a robust phylogenomic backbone. While the broad strokes of the phylogenetic relationships in and of streptophytes had been settled and cemented starting a bit more than 10 years ago (Wodniok *et al*., 2011; Wickett *et al*., 2014; Puttick *et al*., 2018), research into streptophyte algal trait evolution has suffered from low phylogenomic resolution within its major lineages.

**Fig. 4:**
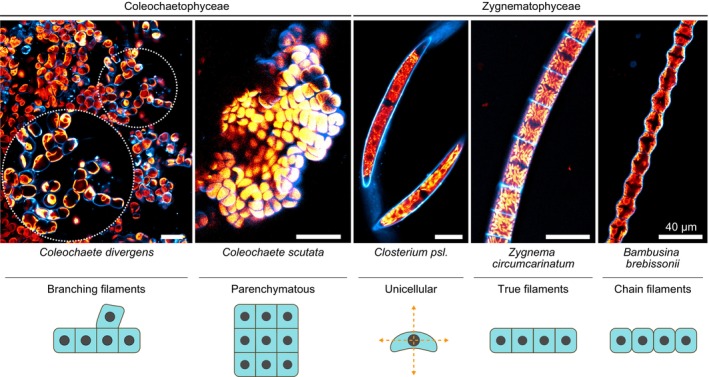
Variation in body plan complexity among the closest and second closest algal relatives to land plants. Confocal laser scanning micrographs of representative Coleochaetophyceae (*Coleochaete divergens* SAG 32.97 and *Coleochaete scutata* SAG 2665) and Zygnematophyceae (*Closterium peracerosum–strigosum–littorale* NIES‐68, *Zygnema circumcarinatum* SAG 698‐1b and *Bambusina brebissonii* SAG 20.82). Chlorophyll *b* autofluorescence is gradient colored in orange/red and calcofluor white‐based fluorescent staining of the cell wall in teal; the micrograph of *C. scutata* was generated by z‐stack imaging and the 3D view was computed from 40 individual optical slices. All pictures were taken by T. Darienko, C. F. Kunz and J. de Vries.

Now, phylogenomic frameworks providing within‐class resolution have been established (Irisarri *et al*., 2021; Hess *et al*., 2022; Bierenbroodspot *et al*., 2024a, 2025; Baştürk *et al*., 2026). Combining a phylogenomic backbone with molecular clock analyses and ancestral character state reconstruction, the concerted outcome of these efforts was that the origin of streptophyte multicellularity likely occurred more than 1 billion years ago (Bierenbroodspot *et al*., 2024a). Across the trajectory of streptophytes belonging to Klebsormidiophyceae and Phragmoplastophyta, we would predict multicellular ancestors (Bierenbroodspot *et al*., 2024a; Fig. 1). And assuming multicellular ancestors, the lineage of Klebsormidiophyceae reverted from filamentous growth back to unicellularity multiple times – as lineage‐specific changes (Bierenbroodspot *et al*., 2024a). From a multicellular ancestor, the aforementioned gains in: 1) charophyceaen complexity occurred at the base of the crown‐Charophyceae; and (2) the discoidal Coleochaetophyceae emerged as a group from (likely branched) filamentous ancestor algae (Bierenbroodspot *et al*., 2025; Figs 1, 4). How is this recurrent secondary gain of body plan complexity to be explained (Niklas & Newman, 2013; Niklas & Newman, 2020; Fig. 3)? Once rolling, the gain of cell–cell connections sets a ratcheting effect in motion, entrenching cellular dependencies through noncell autonomous effects. Thus, the tape of evolution is not replayed anew but follows the same tact (Fig. 3). In the Zygnematophyceae, something astounding happened. From an ancestor that by all accounts we must assume was multicellular, likely a branching filament (Delwiche & Cooper, 2015; Fürst‐Jansen *et al*., 2020), the Zygnematophyceae ancestor possibly reverted to unicellularity (Hess *et al*., 2022; Fig. 4). And even if such unicellularity was not ancestral, the deep genetic backbone of Zygnematophyceae is littered with inferred unicellular ancestors (Hess *et al*., 2022). One of the outstanding questions is how much of – and on what levels – the multicellularity toolkit was retained by unicells (Zegers *et al*., 2024; Goldbecker & de Vries, 2025).

Thus, the patterns underpinning streptophyte body plans are complex and difficult to contextualize along streptophyte evolution. How to move forward in better understanding the evolution of streptophyte multicellularity given this complexity? The answers can be manifold. But we argue that the possible ways forward boil down to understanding the concerted action of regulation and decision‐making at the cellular level and determining what the fundamental morphogenetic force might be.

## III. A preamble on trait complexity and regulation

Streptophyte algae share a significant fraction of their molecular cell biology with that of land plants. And here, especially the zygnematophytes stand out. As such, their cells are streamlined machines that can inform us on the fundamental processes that shape plant biology (Goldbecker & de Vries, 2025; Carrillo‐Carrasco *et al*., 2025a). Multicellularity is a complex trait, requiring the concerted action of diverse cellular mechanisms. This is reflected in the actualization of multicellularity traits themselves; for example, zygnematophytes can divide merely by cleavage and under the co‐occurrence of both phragmoplast and cleavage (Fowke & Pickett‐Heaps, 1969a, 1969b; Marchant & Pickett‐Heaps, 1973; Sawitzky & Grolig, 1995; Buschmann & Zachgo, 2016); we will elaborate on this in much greater detail in the Section VI. It is likely that the formation of plasmodesmata is tied to the mechanisms that act during cytokinesis – including phragmoplast formation; indeed both phragmoplast and plasmodesmata formation emerged as co‐occurring processes at the base of Phragmoplastophyta (Tee & Faulkner, 2024). But the homology of neither the plasmodesmata nor the phragmoplasts found across the diversity are clear. It is also not clear to what degree the formation of a phragmoplast and plasmodesmata are exactly linked. Furthermore, during the course of evolution, the formation of primary plasmodesmata might have reverted to solely forming secondary plasmodesmata in Coleochaetophyceae (Wegner *et al*., 2023) – which would be in line with reduction down to simple filament growth forms from which the ability to grow as complex branched filaments re‐emerged (Bierenbroodspot *et al*., 2025). The same applies for multicellularity itself, which has been lost multiple times over the tree of streptophytes, reverting back to unicells (Bierenbroodspot *et al*., 2024a) and/or multiple transitions between the forms (Hess *et al*., 2022). Yet, the recurrent emergence together with a likely conserved set of genes for morphogenetic cell division across unicells and multicells makes it conceivable that parallel evolution is at work. In this regard, two observations affect our inferences: (1) in the very distantly related seaweeds, similar (albeit difficult to pinpoint) molecular mechanisms bring about macrophyte complexity (Goldbecker *et al*., 2024; Nelson *et al*., 2024); (2) multicellularity could be both a labile but also a plastic trait, as simple salt stress can induce filament formation in unicellular zygnematophyte and a reversion to unicellularity in a filamentous one (Zegers *et al*., 2026a).

The genes coding for the proteins orchestrating cell division processes that underpin morphogenesis are found across diverse streptophyte algae (Nishiyama *et al*., 2018; Dadras *et al*., 2023; Feng *et al*., 2024; Bierenbroodspot *et al*., 2025). In fact, their presence or absence alone does not draw a clear‐cut line between unicellular and multicellular streptophyte algae (Feng *et al*., 2024) – which is not surprising since the lack of a clear genotype‐based distinction between unicellular and multicellular algae was also noted for chlorophytes (Prochnik *et al*., 2010; Umen & Herron, 2021). Fortunately, genes that concertedly act in cell division, cell growth and cell wall modification are easily recovered from co‐expression analyses of streptophyte algae (Dadras *et al*., 2023, 2025; Rieseberg *et al*., 2025). Applying a time‐course analysis of decisive stages in cell division has the potential to unveil which cohorts of genes act to bring about the consecutive steps that underpin the decision‐making in unicellular vs multicellular cell division.

The cell is a clockwork, and when a cellular process is initiated, it determines a gross outcome for the organism as a whole. Cell division processes are the archetype of such a clockwork. Regulated by the conserved action of periodical expression of cyclins and phosphorylation by cyclin‐dependent protein kinases (Inze & De Veylder, 2006), it brings about the ticking of the clockwork. Yet, is this ticking what determines the ultimate morphogenetic decision‐making?

In plants with macroscopic bodies, there is the century‐old to and fro between two prevailing views: the cellular and the organismic theory (Schleiden, 1838; Schwann, 1839; Kaplan & Hagemann, 1991). The former follows a reductionistic rationale, arguing that the whole body can be understood best by dissecting its single components – the cells, and that their concerted and collaborative action underpins all functions. The latter states that the whole body is more than the sum of its parts, becoming an entity able to differentiate and act that is only understandable due to their synergic action. Morphogenic biophysical effects result from tissue tension – the backward‐acting information that stems from tissue tension and is informative to cellular behavior (Woudenberg *et al*., 2025). With the zygnematophytes as closest algal relatives of land plants – invariably complex multicellular organisms – we have a unique opportunity (Goldbecker & de Vries, 2025): to use this system to disentangle these two views in fully operational organisms that frequently transitioned between unicellular and multicellular body plans (Hess *et al*., 2022). How do filamentous zygnematophytes, some of which can branch (Stancheva *et al*., 2014; Buschmann, 2020), integrate noncell‐autonomous positional information and cell context in the absence of, for example, communication via plasmodesmata? Is it a biophysical feedback mechanism? Which aspect of this information exchange is present in unicells? Investigating fundamental decision‐making processes in single cells will yield informative insights into the routes that embryophytic multicellular complexity might have taken when emerging from filamentous branching ancestral algae. In the next sections, we will outline a few such avenues in what could constitute these fundamental mechanisms of streptophyte cells.

## IV. Biophysical feedback mechanisms and sensing of the status of the cell

Streptophyte cells are surrounded by a rigid yet plastic structure – the cell wall. The cell wall does not only shield from the outside and helps maintaining inner integrity, it also serves as a ‘sensor’ of the outside. For this, the cell wall integrity (CWI) is constantly monitored at the cell wall–plasma membrane interface. Using the CWI mechanism, the cell's own status (i.e. cell wall damage and turgor pressure) during growth and in response to the environment is sensed (Hamann, 2015). Owing to this central role, CWI is heavily interconnected with other signaling pathways, including immunity and development (Engelsdorf *et al*., 2018; Vaahtera *et al*., 2019).

Seminal work on the signaling mechanisms of CWI was carried out in yeast – a unicellular organism. The monitoring of CWI has been categorized in three different groups: (1) perception of cell wall damage; (2) mechanical stress (i.e. by membrane stretching); and (3) osmotic stress (Hamann, 2015). In multicellular embryophytes, the CWI mechanism follows the same principles as in the unicellular yeast: perception at the plasma membrane with membrane bound receptors, activation of mechanosensors, influx of Ca^2+^, signal relay using CALCIUM‐DEPENDENT PROTEIN KINASEs and MITOGEN‐ACTIVATED PROTEIN KINASEs as well as transcriptional reprogramming (Bacete & Hamann, 2020). Some processes are even governed by homologous proteins in yeast and plants (Nakagawa *et al*., 2007). While streptophyte algae lack some of the orthologs that regulate CWI signaling in plants, they still contain the components necessary to monitor and modulate their cell walls (Goldbecker & de Vries, 2025; Kunz *et al*., 2026; Zegers *et al*., 2026b).

What is the difference between sensing the cell wall in a unicellular vs in a multicellular context? One major difference is that in multicellular organisms the cell needs to be able to distinguish between the outside and neighboring tissue. They need to switch from an interface that protects to an interface that connects. Triggers for this are likely of an environmental and/or cell–cell communicational nature that might also hinge on components of the CWI system.

Another aspect of multicellularity is the aforementioned mechanical constraints that tissues impose. In multicellular embryophytes, mechanical forces derived from cell wall stiffness as well as the tissue context influence developmental processes, that is via the orientation of microtubules (Hamant *et al*., 2008; Woudenberg *et al*., 2025). This process also acts in the orientation of cell division (Louveaux *et al*., 2016; Blanchard *et al*., 2024). Mechanical constraints are likely to play a role in the development of disk‐shaped *Coleochaete* and might even influence filamentous members of the streptophyte algae.

In land plants as well as Coleochaetophyceae and Charophyceae, neighboring cells are connected with plasmodesmata (Brunkard & Zambryski, 2017; Wegner *et al*., 2023), which clearly distinguishes the cell walls facing the outside and cell–cell connections. While plasmodesmata serve the intercellular communication (and thereby tissue or even organ polarity), they are not the only means of achieving this. In the zygnematophyte *Zygnema* as well as the klebsormidiophyte *Klebsormidium nitens*, which do not possess plasmodesmata, there are differences in cell wall composition and polar localization of cell wall modifying enzymes (Herburger & Holzinger, 2015; Herburger *et al*., 2018; Feng *et al*., 2024). Also, we may expect some form of intercellular communication in these simple filaments, since additional relevant mechanisms may be at play, including signaling based on auxin and LEUCINE‐RICH REPEAT RECEPTOR KINASEs. While the distinctions between ‘protection and connection interfaces’ exist in streptophyte algae (even filamentous ones), we do not know which signals drive this polarization – and it is unclear how these switches can be reverted. But it is clear that polarization is at the heart of several key processes in the physiology and development of streptophyte algae – even the unicellular ones.

## V. Polarity: a molecular nexus in complexity of streptophyte cell biology and physiology

Cell polarity is key for multicellular growth. It enables cells to establish spatial asymmetry, which is essential for organizing and coordinating development. In *Marchantia polymorpha*, for example, the spores establish polarity before the initial division, which occurs asymmetrically to define the orientation of the developmental axes into apical germ cell for the plant body and the smaller basal cell for the germ rhizoid cell (Attrill *et al*., 2024; Roetzer *et al*., 2026). This establishment of asymmetry is dependent on blue‐light perception by the blue‐light photoreceptors PHOTOTROPINs (PHOT) followed by signaling via NPH3/RPT2‐like (NRL) PROTEIN FOR CHLOROPLAST MOVEMENT 1 (NCH1) rather than sensing of gravity (Roetzer *et al*., 2026). While the blue light is the cue for the asymmetric organization of the spore cell, the mechanism by which this asymmetry is established is by the microtubule‐ and actin‐mediated movement of the nucleus to the basal pole. The cytoskeleton reorganization provides both direction and force for the nuclear movement (Miller & Greany, 1974; Vogelmann *et al*., 1981; Buschmann *et al*., 2016; Attrill *et al*., 2024; Roetzer *et al*., 2026). Interestingly, while in *M. polymorpha* the establishment of an asymmetrical division is dependent on blue light, it has been shown that blue light inhibits spore germination in free‐sporing plants (Suo *et al*., 2015). Similarly, cell division in *Marchantia* is stimulated by additional parts of the light spectrum, such as red light, but these wavelengths do not establish polarity, whereas the formation of asymmetry in flowering plants, such as *Arabidopsis thaliana* is dependent on low‐intensity red light perception by Phytochrome (PHY) B and its downstream signaling. The mechanism of this polar expansion is again mediated by microtubule arrangement (Cho & Choi, 2025).

The establishment of cell polarity is central to diverse processes in streptophytes: It governs cell division, sexual reproduction–related development and the coordination of slime secretion for directed movement. Yet, one of the most palpable polar patterns is the production and site‐specific deposition of diverse – and likely diversely sourced (Davies *et al*., 2022; Kunz *et al*., 2024a; Kunz *et al*., 2026) – ultraviolet (UV)‐protective compounds that provide a natural polarity staining. In some zygnematophytes, the whole cell (within a filament) is filled with these compounds (Remias *et al*., 2012b; Aigner *et al*., 2013; Procházková *et al*., 2025). In others, the cell exports the pigments in a highly directional manner to the site facing the irradiation source (Busch & Hess, 2021). How is this unilateral directionality perceived and answered upon? The answer will likely be the aforementioned photoreceptors – in most plants, blue light monitored by PHOT and, additionally, in diverse plant and algal groups, red light monitored by PHY (Goyal *et al*., 2013; Hart & Gardner, 2021); streptophyte algae additionally boast unique photoreceptors, the neochromes (Suetsugu *et al*., 2005; Li *et al*., 2015). In the zygnematophytes exporting pigments in the direction of incident light, homologs to all photoreceptors are present, and the red light detecting phytochromes were upregulated upon UV‐B exposure (Busch *et al*., 2024).

But how is this detection of incident light translated into site‐specific deposition of photoprotective compounds? The production of these photoprotective compounds is induced by UV‐B light, so either the UV photoreceptor UVR8 detects the directionality of light, or it is a concerted collaboration between multiple photoreceptors, where some detect the light source directionality as well as quality, and others, like UVR8, only initiate UV responses, which then build upon previously established systems directing the compounds produced to the site of need. How would such systems leading to site‐specific trafficking be established? Or is the directionality of pigment export created by specific targeting of the involved transporters? Polar proteins and mechanosensing of tension between the plasma membrane and cell wall are involved in the correct targeting of PINFORMED1 (PIN1), which is crucial for its functionality (Gorelova *et al*., 2021; Luschnig & Friml, 2024). The transporters responsible for pigment export could also be targeted to specific membrane microdomains by post‐translational modifications. Is there some biophysical change within the membrane microdomains closest to the incident light? Is the membrane potential or apoplastic pH changed locally by the incident light, creating a gradient that informs the movement of pigments or transporters to the site of need? Is the reorganization of the cytoskeleton involved, along which vesicles with the pigments are transported, maybe even with nuclear movement away from the site of incident light, as observed in *Marchantia* with blue light (Attrill *et al*., 2024; Roetzer *et al*., 2026)? Hence, the question remains: What is the underlying mechanism which enables the targeted deposition of compounds protecting from incident light?

A big player in land plant development is the signaling molecule auxin (Vanneste *et al*., 2025), whose polar distribution throughout the developing plant body also correlates with mechanical forces (Landrein *et al*., 2015). In embryophytes, auxin is trafficked between cells using the polarly distributed PIN transporters (Müller *et al*., 1998). The streptophyte algae *Klebsormidium nitens* have a functional PIN auxin efflux transporter, which, however, does not localize polarly in heterologous expression experiments (Skokan *et al*., 2019). Complementation assays with *Chara braunii* PIN transporters could not recover auxin trafficking and, like the *Klebsormidium* orthologs, showed no polar localization (Kurtovic *et al*., 2025). Auxin trafficking via PINs could be an ancient process, but their polar localization seems limited to embryophytes.

Streptophyte algae do not possess the canonical nuclear auxin response pathway (Mutte *et al*., 2018; Blazquez *et al*., 2020; Carrillo‐Carrasco *et al*., 2023). Yet, they show a phenotypic growth response when treated with auxin (Wood & Berliner, 1979; Ohtaka *et al*., 2017; Kurtovic *et al*., 2025). However, this response might not be auxin‐specific, as it could be phenocopied by tryptophane treatment in the zygnematophyte *Penium* (Carrillo‐Carrasco *et al*., 2025b). Although the transcriptional response to auxin treatment is not conserved between land plants and streptophyte algae, a streptophyte‐wide phosphorylation response to auxin involving RAF kinases was recently discovered (Kuhn *et al*., 2024; Kurtovic *et al*., 2025).

While auxin is a conserved developmental signal, polar auxin trafficking via PIN proteins does not appear to be essential for multicellularity in streptophyte algae. Auxin might have a yet unknown ancestral function that gets co‐opted in some multicellular body plans, as it also influences the development of the distantly related brown algae (Bogaert *et al*., 2022). It will be interesting to determine the extent to which mechanical forces or auxin influence the multicellular streptophyte algae body, which developmental programs exist in the algae, and to what extent these are conserved with land plants.

Overall, different biological programs can be superimposed onto cellular polarity, including localized deposition of cell wall material, the accumulation of specialized protective compounds and the formation of localized outgrowths or protrusions. For the directed and polar response to light, the cell needs to identify and target back to certain locations within the cell. We would argue that the establishment of polarity within a single cell is providing the chassis for what occurs in a multicellular organism – instead of biomolecules and compounds like phytohormones being differentially concentrated in different cells, all of this occurs intracellularly. Such intracellular and noncell‐autonomous mechanisms could be evolutionarily related, providing a possible answer to how a molecular chassis might be retained that: (1) allows for the repeated transitions to and fro uni‐ and multicellularity in zygnematophytes; and (2) constitutes fundamental homologous mechanisms that act in both single‐celled zygnematophyte and complex land plant bodies (see also Goldbecker & de Vries, 2025). Elucidating the molecular nexus around polarity thus has the potential to shed light on the evolution of diverse mechanisms like cell differentiation in multicellular organisms.

## VI. Dividing the cell: a concerted cell biological process

Cell division is at the heart of the morphogenetic process and requires the coordination of the endomembrane system by the cytoskeleton to produce a new cell wall. The cells of land plants are known to divide centrifugally using a cell plate produced by a phragmoplast. Before cell division a preprophase band of microtubules (PPB) is formed. Centrosomes with centrioles are never found. Very different is the assumed mode of cell division in the streptophyte ancestor. Trait reconstruction suggests that the original mode of cell division was centripetal cleavage. Centrosomes with centrioles and astral microtubules were formed here, reminiscent of animal mitosis. A similar type of cell division is seen today in sarcinoid *Chlorokybus* (Lokhorst *et al*., 1988; Fig. 2c) and, likely, in single‐celled *Mesostigma* (Rogers *et al*., 1981; Fig. 2a). The mode of cell division was subject to pronounced evolutionary change in the streptophyte lineage. Cleavage was eventually replaced by the phragmoplast. Centrosomes got lost – maybe because they were not as flexible as their animal counterparts due to the lack of cytoplasmic DYNEIN1 (Schmit & Nick, 2008; Buschmann & Zachgo, 2016). The PPB is a novelty of embryophytes (including the PPB‐protein TANGLED (Muller *et al*., 2006; Walker *et al*., 2007)), although the PPB may have a similar, potentially homologous, structure in the Zygnematophyceae (termed isthmus microtubule band here). The Zygnematophyceae show another oddity in respect to cell division: at least some filamentous species like *Spirogyra* and *Mougeotia* appear to form a phragmoplast which, however, is only involved in the final stages of cytokinesis. Here cytokinesis is initiated by an inward growing septum, indicative of the re‐establishment of the cleavage mechanism. Concomitant with evolutionary changes in cell division, streptophytes also brought forth different types of simple and complex multicellularity (Fig. 3). It was speculated before that the evolutionary change in cell division, especially the introduction of the phragmoplast, enabled a rotation of the plane of cytokinesis, which in turn may have enabled filament branching (based on T junctions) and even tissue formation (Pickett‐Heaps *et al*., 1999). While this is conceptually attractive, it is noteworthy that cell packets as seen in *Chlorokybus* or *Interfilum* can be interpreted as being produced through a rotation of the division plane (Mikhailyuk *et al*., 2018; Bierenbroodspot *et al*., 2024a). How these cell packets are formed precisely awaits further investigation.

It is conceivable that, in its infancy, the streptophyte lineage consisted of flagellated unicells from which sarcinoid cell aggregates emerged (Figs 2, 3); extant *Mesostigma* and sarcinoid *Chlorokybus* exemplify both. One thus could assume that sarcinoid cell packages were the first multicellular assemblies – instead of filaments. However, one can also envision a trajectory from single cells to filaments, and single cells to sarcinoid. In sarcinoid multicellularity, the external (maternal) cell wall might be the essential glue, while in filaments, cell‐to‐cell adhesion might play a pivotal role. Filamentous Klebsormidiophyceae, like *Klebsormidium nitens* that divide by cleavage within the filament (no apical growth), may be considered the prototype of simple filamentous multicellularity in streptophytes. Is cell division fundamentally different if the daughter cells are to be separated, or whether they stay together in, for example, a filament? It is reasonable to assume the original single‐celled streptophyte alga had a midbody‐like structure and used ECRTIII for the final stages of cytokinesis (Mayer & Jürgens, 2004; Niklas, 2014). If the cell cycle is accelerated relative to the time needed for abscission after cytokinesis, multicellularity is readily achieved and related effects have been observed concerning experimental multicellularity in yeast (Bozdag *et al*., 2023). The permanent linkage of cells requires a resistance to tension induced by the environment; therefore, specific mechanisms to achieve adhesion are required. In theory, such adhesion could be achieved by co‐opting genes and mechanisms previously involved in substrate attachment (if present). Considering what we have learned from land plant cells, we may posit that cellular adhesion in streptophyte algae is achieved by a middle lamella‐like structure, involving pectins, arabinogalactan proteins (AGPs) and Ca^2+^. Ca^2+^ acts as a ‘cellular glue’ in other eukaryotes as well, for example via the membrane protein cadherin in the cell–cell adhesion of animals or the glycoprotein‐based adhesion known from fungi (Niklas, 2014). In land plants, much information is available on how fruit ripening, fruit dehiscence, petiole abscission, and more, is regulated at the cellular level. It seems therefore justified to speculate that in streptophyte algae the decision as to whether cells stay together or not is executed by acting on the pectin matrix, interfering with AGP function or manipulating the binding of Ca^2+^ to the cell wall (Roberts *et al*., 2002; Jarvis *et al*., 2003; Pelletier *et al*., 2010); enzymes involved in such processes might hinge on what has been collectively referred to as autolysins and some isoforms are known from *Chlamydomonas* (more below). It remains to be determined whether such mechanisms act in evolutionary lability through gains and losses of multicellularity (Hess *et al*., 2022) and/or the plasticity of multicellularity, where a species can grow as unicell or filamentous depending on the environmental conditions (Zegers *et al*., 2026a), in zygnematophytes.

Complex multicellular organisms display not only adhesion between daughter cells but also intercellular communication and 3D‐tissue differentiation. As a consequence, only certain cells have direct contact with the environment (Knoll, 2011), we observe the division of labor (between cell or tissue types) and some or many cells of the thallus will not reproduce but eventually die. In the complex macrophytes *Chara* and *Nitella* (Charophyceae), complicated cell division patterns create the so‐called node, which is also the basis of thallus branching (Cook *et al*., 1998; Buschmann, 2020). The term ‘complex multicellularity’ may be applied to certain Coleochaetophyceae as well, while the Zygnematophyceae, with their many reductions, appear to have returned to simple multicellularity (or even unicells). In complex multicellularity, the phragmoplast plays a central role as it is involved in rotating the plane of cell division. A phragmoplast is not seen in unicellular streptophytes, nor in most chlorophytes – some of which, however, form a phycoplast that is not considered homologous.

When zygnematophycean algae return to the unicell stage, the phragmoplast is lost (Buschmann & Zachgo, 2016). In other words, complex multicellular development is a hallmark of a *bona fide* phragmoplast mechanism. Another way in which mitosis and cytokinesis may contribute to complex multicellularity are via asymmetric cell divisions. Asymmetric cell divisions produce cell clones with distinguishable developmental fate. New cell types are produced in this way and this can, if cells continue to divide, serve the patterning of tissues and even organ formation (Pillitteri *et al*., 2016). Asymmetric cell divisions are found in streptophytes with phragmoplasts: They are well‐described in Charophyceae and Coleochaetophyceae and the embryophytes (Foissner & Wasteneys, 2014); furthermore, asymmetric cell divisions are the basis of rhizoid formation in *Mougeotia* and related Zygnematophyceae. One could consider this an echo of the complex multicellularity lost in these algae (Buschmann, 2020), reflecting the deeply conserved morphogenetic toolkit of phragmoplastophytic streptophytes. While extant Zygnematophyceae exhibit a deep genetic structure characterized by several ancient radiations from the last common ancestor (LCA) of the class (Hess *et al*., 2022; Bierenbroodspot *et al*., 2026), the lack of a robust palaeobotanical record makes it impossible to infer what extinct stem zygnematophyceans may have looked like; in fact, in light of our views presented here we consider it likely that branching was a much more common phenomenon in certain extinct groups of stem Zygnematophyceae (Fig. 1). Yet, also extant Zygnematophyceae are more complex than might superficially appear, to which we will turn next.

## VII. Unique events and unicellular complexity shape the evolution of the Zygnematophyceae

Considering the trajectory of phragmoplastophyte evolution, it is most likely that the common ancestor of the Zygnematophyceae and Embryophyta had the following features: It was filamentous with plasmodesmata as cell–cell connections, producing flagellated cells with unilateral basal body orientation (probably with MLS). This branching ancestor was presumably capable of cell differentiation and exhibited apical growth. Oogamous sexual reproduction likely took place under the influence of species‐specific pheromones. The life cycle was haplontic (or perhaps even isomorphic haplo‐diplontic, Renner & Sokoloff, 2024). All these features are present in the Charophyceae and the Coleochaetophyceae, with different modifications. However, many of these characters are lost in species of Zygnematophyceae (Fig. 1). What fostered this reduction is unclear. There are speculations that the conditions of Snowball Earth fostered the evolution of unique (one direction) features (Zarsky *et al*., 2021) – for which there are compatible (Bowles *et al*., 2024b) and noncompatible data (Bowles *et al*., 2024a). The major evolutionary event was probably that the common ancestor of the Zygnematophyceae lost the ability to produce flagellated cells, which had the biggest impact on the further evolution of this group. As a consequence, almost all cells (except the rhizoid cell structures found in many genera, such as *Mougeotia*, *Zygogonium* and *Spirogyra*; Ikegaya *et al*., 2008) remained omnipotent, with fragmentation being the only possible form of asexual reproduction (either passively through mechanical break‐up or actively under the influence of autolysins). This may have allowed the genes required for the formation of plasmodesmata to evolve under relaxed constraints. Simultaneously, a whole range of new complex features came about in zygnematophytes. A new mode of sexual reproduction evolved: conjugation. The lack of flagellated cells probably fostered the evolution of ornamented cell walls with pores for excretion of mucilage in Desmidiales, which allowed the cells to move. By production of pheromones, sexual compatible cells were able to move together for conjugation. Another strategy evolved in the filamentous taxa. Growing in large mats floating on surfaces of ponds and lakes allows them to find a compatible partner for conjugation. For example, Kadlubowska (1984) described that *Spirogyra fluviatilis* grows vegetatively benthic, but forms floating mats for conjugation. However, this restricted those taxa to such aquatic habitats. The reduction from filaments to unicells is also reflected in differences during cell division. Whereas the filamentous taxa divide by a unique combination of cleavage with a finalizing phragmoplast, unicells, such as *Cosmarium* or *Closterium* have returned to cleavage (Buschmann & Zachgo, 2016).

Traditionally, Zygnematophyceae were described as placoderm or saccoderm, being taxonomically classified into Desmidiales and Mesotaeniales, respectively. Placoderm taxa consist of two semi‐cells of different age often separated by a visible isthmus where the nucleus is located. Cell walls are ornamented and often have pores. By contrast, the cells of saccoderm taxa are not divided in semi‐cells and lack an isthmus. Based on the current understanding of the Zygnematophyceae phylogeny, all placoderm taxa belong to the Desmidiales whereas saccoderms are distributed across Serritaeniales (*Mesotaenium endlicherianum*, *Serritaenia* ssp.), Zygnematales (*Cylindrocystis*, other species of *Mesotaenium*) and Spirogloeales (Busch & Hess, 2022). The cell division of the saccoderm *Netrium digitus* is similar to that in the order Desmidiales (Pickett‐Heaps, 1975). The composition of the cell walls is quite complex, especially among the unicellular zygnematophytes. Three layers can be observed, a hyaline or amorphic outer layer and two fibrous primary and secondary inner layers (Mix, 1972, 1973). Considering its structures, three types of cell walls were recognized: *Mesotaenium*‐types, *Penium*‐types and *Cosmarium*‐types, which differ in the segmentation, incrustation and the presence/absence of pores. The cell wall of type 1 (*Mesotaenium*) consists of one piece with a smooth hyaline outer layer without pores and incrustations. It can be found in the orders Serritaeniales, Zygnematales, Spirogyrales and Spirogloeales. The types 2 (*Penium*) and 3 (*Cosmarium*) are present only in the Desmidiales. These cell walls are segmented in several (type 2) or two pieces (type 3). The outer layers are very structured with warts, spines and strips with incrustations and pores. The excretion of mucilage through these pores allowed the desmids to move as reaction to changing light conditions (Häder & Wenderoth, 1977; Lütz‐Meindl & Brosch‐Salomon, 2000). The complex chloroplast shapes of zygnematophytes shows the highest diversity among green algae. To what degree this is linked to modes of cell division is unclear, but in *Spirogyra*, which lacks almost all canonical plastid division genes, the current idea is that plastid division is executed at the time of cytokinesis (Goldbecker *et al*., 2025). The unicellular taxa have mostly two chloroplasts of different shapes, whose morphology is dependent on the cell shape. The chloroplasts of most species contain pyrenoids with the exception of *Mougeotiopsis*. *Mougeotia* is able to react to changing light conditions with the rotation of the plate‐like chloroplast (Haupt, 1959). While the Desmidiales are best known as ornamented unicells, they exhibit a form of filamentous organization by growing into connected chains of cells (e.g. *Bambusina*; Fig. 2g). This may constitute an even further simplified form of multicellularity; however, cross wall ornamentation in these cells can be quite varied, perhaps indicating species‐dependent modifications of the centripetal cleavage mechanism seen in these cells (Hall *et al*., 2008).

As mentioned above, the asexual reproduction differs among the Zygnematophyceae. Cell division, especially in desmids, differs from all other streptophyte algae. Cell division here is based on semi‐cells that are produced at the time of mitosis/cytokinesis. After division of the nucleus, the protoplasts extend through the cell wall opening at the isthmus by turgor pressure, followed by production of stretchable cell wall material of the primary cell wall (Meindl, 1993; Felhofer *et al*., 2021). The younger semi‐cell increases up to the size of the first half. The chloroplast divides and moves through the isthmus into the younger semi‐cell. More cell wall material is produced until the typical ornamentation of the cell is finished and the two cells are finally separated (Kiermayer, 1970). Filamentous zygnematophytes can propagate via fragmentation, which is an active process triggered by the excretion of self‐degrading enzymes that dissolve the traverse cell walls of filaments. These enzymes (autolysins) can be strain/species‐ or even stage‐specific as demonstrated for the chlorophyte genera *Chlamydomonas*, *Geminella* and *Uronema* (Schlösser, 1976, 1981, 1987). In *Chlamydomonas*, three autolysins are known: vegetative lytic enzyme, which only lyses sporangia and is species‐specific, gamete lytic enzymes, which lyse all cell stages except zygotes and are species‐group specific, and zygote lytic enzymes, which lyse the zygotes after meiosis (Schlösser, 1976, 1984; Matsuda, 1988). Autolysins of *Chlamydomonas* are Zn‐metalloproteases and their genes have been identified (Matsuda *et al*., 1985; Kubo *et al*., 2009). For filamentous zygnematophytes, the process of fragmentation has been intensively studied by Benecke (1898) and the process was cinematically documented for *Zygnema circumcarinatum* by Schlösser & Renkert (1992; https://av.tib.eu/media/14346). That said, little is known about the underpinning molecular biology. Fragmentation was also observed in *Klebsormidium*, but the participation of autolysins needs further investigation. Until now, the genes coding for autolysins are unknown in streptophytes but could be related to genes involved in abscission in land plants (as described in the previous section).

The *Closterium peracerosum–strigosum–littorale* complex is a model for sexual reproduction among Zygnematophyceae (Tsuchikane & Sekimoto, 2019; Ohtaka & Sekimoto, 2023); in particular to understand the complex cell biology of conjugation. This reproductive process involves the fusion of two compatible *Closterium* cells. Conjugation happens through several stages: cell division, pairing of compatible cells, formation of conjugation papillae, release of gametic protoplasts and cell fusion to form a zygospore (Tsuchikane *et al*., 2010; Sekimoto *et al*., 2012). Conjugation depends on precise cell–cell recognition, and evidence suggests that dynamic changes in *Closterium* cell surface carbohydrates – particularly during sexual reproduction – are essential for gamete recognition, adhesion and fusion, with multiple carbohydrate types likely involved (Hori *et al*., 2012), as well as the conjugation papillae, formed by localized secretion of wall materials, function as adhesion sites that facilitate recognition and physical contact between mating partners (Abe *et al*., 2016). Glycoproteins are crucial in regulating these interactions, acting as recognition and adhesion molecules that determine mating compatibility and initiate conjugation (Hori *et al*., 2012; Tsuchikane & Sekimoto, 2019). They likely operate in conjunction with pheromonal signaling, which induces morphological and biochemical changes necessary for successful gamete fusion (Tsuchikane *et al*., 2005; Akatsuka *et al*., 2006; Tsuchikane & Sekimoto, 2019). These pheromonal cues promote chemotactic attraction and coordination between opposite mating types, ensuring efficient pairing and zygospore development. The genome size of *Closterium* is notably variable as well, partially attributed to correlations between genome size and cell dimensions, which have been documented in related species, such as *Micrasterias* (Kawaguchi *et al*., 2023; Tsuchikane *et al*., 2024). *Closterium* displays a level of cellular organization that differs from that of filamentous zygnematophycean genera, such as *Spirogyra* and *Zygnema*, as well as from simpler unicellular forms like *Mesotaenium* (Pickett‐Heaps, 1975). While filamentous genera express complexity primarily through multicellular organization and cell–cell interactions, *Closterium* exhibits pronounced structural organization within a single cell. Its characteristic crescent‐shaped morphology is associated with polarized growth and a consistent spatial arrangement of organelles, highlighting a form of cellular complexity that operates at the intracellular level rather than through tissue formation (Pickett‐Heaps & Fowke, 1970, 1971; Sekimoto *et al*., 2012). These features make *Closterium* a useful model organism for researching how complex cellular patterns can arise in unicellular streptophytes. Detailed observations on *Closterium* describe the regulated growth polarity and organelle positioning (Pickett‐Heaps & Fowke, 1970). The presence of these traits in unicellular streptophytes supports the idea that key elements of cellular complexity evolved before the emergence of multicellularity in land plants.

## VIII. Connectivity, not complexity?

While the distinction between the emergence of entirely new genes (gain) and the repurposing of existing cellular machinery (tinkering) frames much of streptophyte evolution, a largely unexplored source of evolutionary innovation lies in fine‐scale protein connectivity mediated by short linear motifs (SLiMs, also referred to as eukaryotic linear motifs, ELMs) within intrinsically disordered regions (IDRs; Kumar *et al*., 2024). In human biology, these motifs have been shown to play critical roles in shaping complex interaction networks, and yet they remain poorly characterized in large‐scale interactome studies (Davey *et al*., 2023). In streptophytes, SLiM‐mediated interactions remain mostly invisible. During cell division, for example, most studies have focused on individual protein interactions, while the broader networks linking cytoskeletal elements, polarity factors and cell wall regulators remain largely unexplored (Dahiya & Bürstenbinder, 2023). Detecting these motifs using conventional multiple sequence alignments is extremely challenging because motifs are short, embedded in variable IDRs, and often poorly conserved at the sequence level. Small changes, even single amino acid substitutions, can create new interaction sites or abolish existing ones, leading to rapid, lineage‐specific rewiring of protein–protein interaction networks (Davey *et al*., 2015). Because the outcomes of evolutionary changes are imposed on the crowded interior of the cell, mechanisms that can create new interaction surfaces *ex nihilo* are particularly powerful, providing a rapid path for novel traits to arise.

SLiMs act as modular docking sites for multiple partners. Embedded within IDRs, these motifs confer structural flexibility, allowing proteins to function as scaffolds that assemble macromolecular complexes and adapt to diverse spatial and temporal contexts (From the American Association of Neurological Surgeons *et al*., 2018). IDRs can also drive phase separation and the formation of membrane‐less condensates, which are emerging as key principles of cellular organization (Ibrahim *et al*., 2023; Ozawa *et al*., 2023; von Bülow *et al*., 2025). By concentrating molecules and facilitating dynamic interactions, these proteins can coordinate processes, such as cytoskeletal organization, signaling cascades and cell division. IDR‐containing proteins also provide excellent points for integration of diverse signals through post‐translational modifications, linking environmental and developmental cues to regulatory networks (Bah & Forman‐Kay, 2016; Darling & Uversky, 2018; Dahiya *et al*., 2023). Taken together, SLiM/IDR proteins act as organizers of the cell, shaping structure and function and creating opportunities for novel multicellular traits.

In streptophyte algae, IQ67 Domain (IQD) proteins represent promising candidates for SLiM/IDR‐mediated rewiring of protein networks (Kumari *et al*., 2021). IQDs are largely single‐copy in algal lineages and contain multiple conserved motifs embedded in long IDRs. Variation in motif composition between Klebsormidiophyceae and Phragmoplastophyta suggests early lineage‐specific changes in potential interaction surfaces, while embryophytes later expanded IQDs into three subgroups. These motifs may function as modular docking sites, allowing IQDs to scaffold cytoskeletal regulators, signaling components and cell division machinery (Bürstenbinder *et al*., 2017; Kumari *et al*., 2021; Dahiya & Bürstenbinder, 2023). Their intrinsic disorder likely provides conformational flexibility and could support assembly of macromolecular complexes. Furthermore, the motifs are likely points for integrating diverse signals via post‐translational modifications, linking environmental and developmental cues to regulatory networks. Other protein families appear to follow a similar principle. TON1 Recruiting Motif (TRM) proteins, although detailed phylogenetic analyses in streptophyte algae remain incomplete, contain conserved motifs within IDRs that could enable rewiring of cytoskeletal interactions and integration of phosphatase‐based signaling (Spinner *et al*., 2013; Dahiya & Bürstenbinder, 2023). AIR9, another key regulator of plant‐specific cell division, also harbors IDRs and may help to scaffold microtubule arrays (Buschmann *et al*., 2006). Together, these families suggest that the evolution of multicellular traits in streptophytes may involve rewiring and neofunctionalization of existing protein toolkits rather than solely relying on the emergence of new genes.

Taken together, the potential of IDR/SLiM‐containing proteins to generate new docking sites *ex nihilo*, organize macromolecular complexes, drive phase separation and integrate diverse signals positions them as central organizers of the cell. The genomic stage is set in streptophytes (Kunz *et al*., 2025). Future studies combining motif‐aware comparative genomics (Prieto *et al*., 2024), ancestral interactome reconstruction and functional validation in streptophyte algae will be essential to clarify how these flexible proteins may have contributed to multicellular architectures across streptophytes. The combination of detection difficulty and the potentially profound impact of subtle sequence changes highlights both the challenge and the importance of incorporating SLiM‐mediated rewiring into models of streptophyte evolution. In lineages with frequent evolutionary changes between unicellularity and multicellularity, like Zygnematophyceae and Klebsormidiophyceae (Hess *et al*., 2022; Bierenbroodspot *et al*., 2024a, 2024b), fast evolving molecular protein networks like SLiM/IDR‐mediated signaling hubs could be instrumental.

## IX. Gain vs tinkering

The advent of comparative genomics has largely debunked the idea that evolutionary transitions such as multicellularity are always accompanied by high genomic innovation. In fact, the origin of new genes often predates evolutionary transitions, as is the case for the origin of multicellular animals (Grau‐Bove *et al*., 2017). In plants, the origin of complex multicellularity in Embryophyta is associated with increased gene family sizes through gene duplication (Feng *et al*., 2024). The emergence of new genes in embryophytes is comparable to that inferred for several streptophyte ancestors, being lower than the rates of genomic innovation inferred for the origin of seed plants and angiosperms (Bowles *et al*., 2020). Phylogenomics picture a more complex history of streptophyte multicellularity than previously anticipated, with a billion‐year‐old multicellular ancestor that, during the course of evolution, reverted to unicellularity multiple times, one of the secondarily unicellular ancestors re‐evolved multicellularity repeatedly, and simple multicellular ancestors also evolved more complex parenchymatous body plans. This implies that multicellularity is a highly labile trait that can evolve rapidly among streptophytes. Moreover, some algal strains seem to be able to grow as unicells or as filaments depending on environmental conditions (Bierenbroodspot *et al*., 2024a; Zegers *et al*., 2026a) – a case of phenotypic plasticity. These evolutionary patterns are only possible if we assume an ancient potential for (simple) multicellularity and frequent re‐use of the molecular machinery underpinning multicellular growth. In other words, these transitions are more likely explained by how the pieces of this machinery interact with each other rather than by the emergence of new pieces (Jacob, 1977). This stresses the abovementioned idea that genes do not emerge *de novo* for every new function, but instead, existing genes, regulatory networks and molecular machineries get repurposed during evolution. This reuse is contingent on the genes and interactions that are available in a particular genome, and the outcomes of this repurposing are only as good as needed to pass the genes onto the next generation, as dictated by natural selection. These ideas were beautifully illustrated by François Jacob's (1977) metaphor of evolution being a tinkerer rather than an engineer: It has no plan, depends on available materials and tools and produces good enough (not perfect) outcomes.

Jacob (1977) also pointed out that evolutionary reuse is constrained by organismal complexity: more complex organisms impose additional constraints to the reuse of their parts. In support of this idea, recent examples of irreducible complexity have been identified at the molecular level (Muñoz‐Gomez *et al*., 2021). Perhaps for this reason, we do not know of any reversion to unicellularity within embryophytes. Instead, the transition between unicells and simpler multicellular streptophyte algae is very frequent, suggesting that the molecular switches for these transitions are more easily triggered. The molecular triggers are likely at the regulatory level, given that the genes that govern cell division and communication are conserved across unicellular and multicellular streptophytes. This, together with the observation that multicellular growth can be a plastic response to the environment, suggests the hypothesis that multicellularity might have been a response to environmental challenges that emerged once the building blocks that enable it evolved in streptophyte ancestors, and simple multicellular forms only required a small switch in how these proteins interact with each other after cell division. Therefore, the origins of multicellularity are not only contingent on the genomic background but could also be contingent on the environmental conditions. If the tape of streptophyte evolution were to be replayed (à la Gould, 1989), it would probably lead to multiple instances of multicellularity given the ancestral origin of most implicated molecular machineries, but these might look different from extant multicellular streptophytes due to this environmental contingency.

## X. Conclusion

Streptophytes have the deep genetic potential for multicellularity – up to astounding tissue complexity. The actualization of that potential likely hinges on a combination of factors. A simple gene‐focused research philosophy has failed thus far in determining the key aspects that actualize multicellularity in streptophytes. One aspect will involve gene regulation, aligning with the fact that multicellularity has been gained and lost repeatedly which suggests mechanisms of (relatively) rapid evolution at work; changes in regulation are an apt explanation for such a rapid change – potentially epigenetic and environment‐triggered. Regulatory changes can involve multiple mechanisms like direct transcriptional rewiring but also structural organizations of the cytoskeleton, the action of macromolecular organizers, membrane properties (such as rafts forming a nexus) and more. Another explanation that applies to any streptophyte cell is biophysical feedback loops that act any time cell–cell adjacency emerges. Through such universal cell biological factors, ratcheting effects can propel streptophyte cells – all bearing the apt genetic potential – down the trajectory toward branching filaments with cell–cell dependency. It is such branching filaments that likely represent the body plans at the deep phragmoplastophyte nodes and the LCA of land plants and algae.

## Competing interests

None declared.

## Author contributions

All authors reviewed and discussed the literature and wrote sections of the manuscript. JdV, TD, TP, ML, and MJB drafted section I. JdV drafted sections II, III, and X. ESG drafted section IV. CFK drafted section V. HB drafted section VI. EE, TD, and TP drafted section VII. KB drafted section VIII. II drafted section IX. CFK, ESG, TD, TP and JdV designed the figures. TD, CFK and JdV have taken the micrographs. TD, CFK and ESG, and TP, KB, HB and JdV contributed equally to this work.

## Disclaimer

The New Phytologist Foundation remains neutral with regard to jurisdictional claims in maps and in any institutional affiliations.

## References

[nph71415-bib-0001] Abe J , Hori S , Sato M , Sekimoto H . 2016. Concanavalin A disrupts the release of fibrous material necessary for zygote formation of a unicellular charophycean alga, *Closterium peracerosum‐strigosum‐littorale* complex. Frontiers in Plant Science 7: 1040. 27468295 10.3389/fpls.2016.01040 PMC4942458

[nph71415-bib-0002] Aigner S , Remias D , Karsten U , Holzinger A . 2013. Unusual phenolic compounds contribute to ecophysiological performance in the purple‐colored green alga zygogonium ericetorum (Zygnematophyceae, Streptophyta) from a high‐alpine habitat. Journal of Phycology 49: 648–660. 25810559 10.1111/jpy.12075 PMC4370239

[nph71415-bib-0003] Akatsuka S , Tsuchikane Y , Fukumoto R , Fujii T , Sekimoto H . 2006. Physiological characterization of the sex pheromone protoplast‐release‐inducing protein from the *Closterium peracerosum*‐strigosum‐littorale complex (Charophyta). Phycological Research 54: 116–121.

[nph71415-bib-0004] Attrill ST , Mulvey H , Champion C , Dolan L . 2024. Microtubules and Actin filaments direct nuclear movement during the polarisation of *Marchantia* spore cells. Development 151: dev202823. 39041335 10.1242/dev.202823

[nph71415-bib-0005] Bacete L , Hamann T . 2020. The role of mechanoperception in plant cell wall integrity maintenance. Plants 9: 574. 32369932 10.3390/plants9050574 PMC7285163

[nph71415-bib-0006] Bah A , Forman‐Kay JD . 2016. Modulation of intrinsically disordered protein function by post‐translational modifications. The Journal of Biological Chemistry 291: 6696–6705. 26851279 10.1074/jbc.R115.695056 PMC4807257

[nph71415-bib-0007] Baştürk MN , Kunz CF , Darienko T , Pröschold T , de Vries J , Chen H . 2026. Phylogenomic frameworks from deep archaeplastid evolution to embryophyte diversification. Current Biology 36: R522–R544. 42259268 10.1016/j.cub.2026.03.019

[nph71415-bib-0008] Benecke W . 1898. Mechanismus und Biologie des Zerfalls der Konjugatenfaden in die einzelnen Zellen. Jahrbücher fur Wissenschaftliche Botanik 32: 453.

[nph71415-bib-0009] Bierenbroodspot MJ , Darienko T , de Vries S , Delwiche CF , Lorenz M , Ali Z , Irisarri I , Pröschold T , de Vries J . 2025. Phylogenomics unveil a recent origin of morphological complexity in Coleochaetophyceae. Current Biology 35: 5071–5080. 40967205 10.1016/j.cub.2025.08.046

[nph71415-bib-0010] Bierenbroodspot MJ , Darienko T , de Vries S , Fürst‐Jansen JMR , Buschmann H , Pröschold T , Irisarri I , de Vries J . 2024a. Phylogenomic insights into the first multicellular streptophyte. Current Biology 34: 1–12. 38244543 10.1016/j.cub.2023.12.070 PMC10849092

[nph71415-bib-0011] Bierenbroodspot MJ , Kunz CF , Goldbecker ES , Lorenz M , Irisarri I , Pröschold T , Darienko T , de Vries J . 2026. A deep genetic structure phylogenomically frames the closest algal relatives of land plants. *bioRxiv*. doi: 10.64898/2026.04.20.719584.

[nph71415-bib-0012] Bierenbroodspot MJ , Pröschold T , Fürst‐Jansen JMR , de Vries S , Irisarri I , Darienko T , de Vries J . 2024b. Phylogeny and evolution of streptophyte algae. Annals of Botany 134: 385–400. 38832756 10.1093/aob/mcae091 PMC11341676

[nph71415-bib-0013] Blanchard GB , Scarpa E , Muresan L , Sanson B . 2024. Mechanical stress combines with planar polarised patterning during metaphase to orient embryonic epithelial cell divisions. Development 151: dev202862. 38639390 10.1242/dev.202862 PMC11165716

[nph71415-bib-0014] Blazquez MA , Nelson DC , Weijers D . 2020. Evolution of plant hormone response pathways. Annual Review of Plant Biology 71: 327–353. 10.1146/annurev-arplant-050718-100309 32017604

[nph71415-bib-0015] Blount ZD , Barrick JE , Davidson CJ , Lenski RE . 2012. Genomic analysis of a key innovation in an experimental *Escherichia coli* population. Nature 489: 513–518. 22992527 10.1038/nature11514 PMC3461117

[nph71415-bib-0016] Bogaert KA , Blomme J , Beeckman T , De Clerck O . 2022. Auxin's origin: do PILS hold the key? Trends in Plant Science 27: 227–236. 34716098 10.1016/j.tplants.2021.09.008

[nph71415-bib-0017] Bowles AM , Bechtold U , Paps J . 2020. The origin of land plants is rooted in two bursts of genomic novelty. Current Biology 30: 530–536. 31956023 10.1016/j.cub.2019.11.090

[nph71415-bib-0018] Bowles AMC , Williams TA , Donoghue PCJ , Campbell DA , Williamson CJ . 2024a. Metagenome‐assembled genome of the glacier alga *Ancylonema* yields insights into the evolution of streptophyte life on ice and land. New Phytologist 244: 1629–1643. 38840553 10.1111/nph.19860

[nph71415-bib-0019] Bowles AMC , Williamson CJ , Williams TA , Donoghue PCJ . 2024b. Cryogenian origins of multicellularity in Archaeplastida. Genome Biology and Evolution 16: evae026. 38333966 10.1093/gbe/evae026 PMC10883732

[nph71415-bib-0020] Bowman JL . 2022. The origin of a land flora. Nature Plants 8: 1352–1369. 36550365 10.1038/s41477-022-01283-y

[nph71415-bib-0021] Bozdag GO , Zamani‐Dahaj SA , Day TC , Kahn PC , Burnetti AJ , Lac DT , Tong K , Conlin PL , Balwani AH , Dyer EL *et al*. 2023. De novo evolution of macroscopic multicellularity. Nature 617: 747–754. 37165189 10.1038/s41586-023-06052-1 PMC10425966

[nph71415-bib-0022] Brunkard JO , Zambryski PC . 2017. Plasmodesmata enable multicellularity: new insights into their evolution, biogenesis, and functions in development and immunity. Current Opinion in Plant Biology 35: 76–83. 27889635 10.1016/j.pbi.2016.11.007

[nph71415-bib-0023] von Bülow S , Tesei G , Zaidi FK , Mittag T , Lindorff‐Larsen K . 2025. Prediction of phase‐separation propensities of disordered proteins from sequence. Proceedings of the National Academy of Sciences, USA 122: e2417920122. 10.1073/pnas.2417920122 PMC12002312 40131954

[nph71415-bib-0024] Bürstenbinder K , Moller B , Plotner R , Stamm G , Hause G , Mitra D , Abel S . 2017. The IQD family of calmodulin‐binding proteins links calcium signaling to microtubules, membrane subdomains, and the nucleus. Plant Physiology 173: 1692–1708. 28115582 10.1104/pp.16.01743 PMC5338658

[nph71415-bib-0025] Busch A , Gerbracht JV , Davies K , Hoecker U , Hess S . 2024. Comparative transcriptomics illuminates the cellular responses of an aeroterrestrial zygnematophyte to UV radiation. Journal of Experimental Botany 75: 3624–3642. 38520340 10.1093/jxb/erae131 PMC11156808

[nph71415-bib-0026] Busch A , Hess S . 2021. Sunscreen mucilage: a photoprotective adaptation found in terrestrial green algae (Zygnematophyceae). European Journal of Phycology 57: 107–124.

[nph71415-bib-0027] Busch A , Hess S . 2022. A diverse group of underappreciated zygnematophytes deserves in‐depth exploration. Applied Phycology 3: 306–323.

[nph71415-bib-0028] Buschmann H . 2020. Into another dimension: how streptophyte algae gained morphological complexity. Journal of Experimental Botany 71: 3279–3286. 32270175 10.1093/jxb/eraa181

[nph71415-bib-0029] Buschmann H , Chan J , Sanchez‐Pulido L , Andrade‐Navarro MA , Doonan JH , Lloyd CW . 2006. Microtubule‐associated AIR9 recognizes the cortical division site at preprophase and cell‐plate insertion. Current Biology 16: 1938–1943. 17027491 10.1016/j.cub.2006.08.028

[nph71415-bib-0030] Buschmann H , Holtmannspotter M , Borchers A , O'Donoghue MT , Zachgo S . 2016. Microtubule dynamics of the centrosome‐like polar organizers from the basal land plant *Marchantia polymorpha* . New Phytologist 209: 999–1013. 26467050 10.1111/nph.13691

[nph71415-bib-0031] Buschmann H , Zachgo S . 2016. The evolution of cell division: from streptophyte algae to land plants. Trends in Plant Science 21: 872–883. 27477927 10.1016/j.tplants.2016.07.004

[nph71415-bib-0032] Carrillo‐Carrasco VP , Hernandez‐Garcia J , Girou C , Grubor I , Keller J , Lim E , Schmidt V , Sørensen I , Vosolsobe S , Buschmann H *et al*. 2025a. A roadmap to developing unified streptophyte algal model systems. Current Biology 35: R725–R738. 40695244 10.1016/j.cub.2025.05.023

[nph71415-bib-0033] Carrillo‐Carrasco VP , Hernandez‐Garcia J , Mutte SK , Weijers D . 2023. The birth of a giant: evolutionary insights into the origin of auxin responses in plants. EMBO Journal 42: e113018. 36786017 10.15252/embj.2022113018 PMC10015382

[nph71415-bib-0034] Carrillo‐Carrasco VP , van Galen M , Bronkhorst J , Mutte S , Kohlen W , Sprakel J , Hernandez‐Garcia J , Weijers D . 2025b. Auxin and tryptophan trigger common responses in the streptophyte alga *Penium margaritaceum* . Current Biology 35: 2078–2087. 40209711 10.1016/j.cub.2025.03.037 PMC12061043

[nph71415-bib-0035] Cho S , Choi G . 2025. Phytochrome B regulates cortical microtubule arrangement to control cotyledon polar expansion by repressing LONGIFOLIAs. Plant Physiology 198: kiaf162. 40272438 10.1093/plphys/kiaf162

[nph71415-bib-0036] Clark JW , Hetherington AJ , Morris JL , Pressel S , Duckett JG , Puttick MN , Schneider H , Kenrick P , Wellman CH , Donoghue PCJ . 2023. Evolution of phenotypic disparity in the plant kingdom. Nature Plants 9: 1618–1626. 37666963 10.1038/s41477-023-01513-x PMC10581900

[nph71415-bib-0037] Cook ME , Graham LE , Lavin CA . 1998. Cytokinesis and nodal anatomy in the charophycean green alga *Chara zeylanica* . Protoplasma 203: 65–74.

[nph71415-bib-0038] Dadras A , Duminil P , de Vries S , Irisarri I , Feussner I , de Vries J . 2025. Algal origins of core land plant stress response sub‐networks. The Plant Journal 122: e70291. 40550109 10.1111/tpj.70291 PMC12185132

[nph71415-bib-0039] Dadras A , Fürst‐Jansen JMR , Darienko T , Krone D , Scholz P , Sun S , Herrfurth C , Rieseberg TP , Irisarri I , Steinkamp R *et al*. 2023. Environmental gradients reveal stress hubs pre‐dating plant terrestrialization. Nature Plants 9: 1419–1438. 37640935 10.1038/s41477-023-01491-0 PMC10505561

[nph71415-bib-0040] Dahiya P , Bürstenbinder K . 2023. The making of a ring: assembly and regulation of microtubule‐associated proteins during preprophase band formation and division plane set‐up. Current Opinion in Plant Biology 73: 102366. 37068357 10.1016/j.pbi.2023.102366

[nph71415-bib-0041] Dahiya P , Haluška S , Buhl J , Kölling M , Papsdorf S , Zehnich D , Machalett K , Pfeiffer P , Stamm G , Potocký M *et al*. 2023. Origin and evolution of IQD scaffolds and assembled protein complexes in plant cell division. SSRN Developmental‐Cell‐D‐23‐01058.

[nph71415-bib-0042] Darling AL , Uversky VN . 2018. Intrinsic disorder and posttranslational modifications: The darker side of the biological dark matter. Frontiers in Genetics 9: 158. 29780404 10.3389/fgene.2018.00158 PMC5945825

[nph71415-bib-0043] Davey NE , Cyert MS , Moses AM . 2015. Short linear motifs – ex nihilo evolution of protein regulation. Cell Communication and Signaling: CCS 13: 43. 26589632 10.1186/s12964-015-0120-z PMC4654906

[nph71415-bib-0044] Davey NE , Simonetti L , Ivarsson Y . 2023. The next wave of interactomics: Mapping the SLiM‐based interactions of the intrinsically disordered proteome. Current Opinion in Structural Biology 80: 102593. 37099901 10.1016/j.sbi.2023.102593

[nph71415-bib-0045] Davies KM , Landi M , van Klink JW , Schwinn KE , Brummell DA , Albert NW , Chagne D , Jibran R , Kulshrestha S , Zhou Y *et al*. 2022. Evolution and function of red pigmentation in land plants. Annals of Botany 130: 613–636. 36070407 10.1093/aob/mcac109 PMC9670752

[nph71415-bib-0046] Delwiche CF , Cooper ED . 2015. The evolutionary origin of a terrestrial flora. Current Biology 25: R899–R910. 26439353 10.1016/j.cub.2015.08.029

[nph71415-bib-0047] Donoghue PCJ , Harrison CJ , Paps J , Schneider H . 2021. The evolutionary emergence of land plants. Current Biology 31: R1281–R1298. 34637740 10.1016/j.cub.2021.07.038

[nph71415-bib-0048] Engelsdorf T , Gigli‐Bisceglia N , Veerabagu M , McKenna JF , Vaahtera L , Augstein F , Van der Does D , Zipfel C , Hamann T . 2018. The plant cell wall integrity maintenance and immune signaling systems cooperate to control stress responses in *Arabidopsis thaliana* . Science Signaling 11: eaao3070. 29945884 10.1126/scisignal.aao3070

[nph71415-bib-0049] Felhofer M , Mayr K , Lutz‐Meindl U , Gierlinger N . 2021. Raman imaging of Micrasterias: new insights into shape formation. Protoplasma 258: 1323–1334. 34292402 10.1007/s00709-021-01685-3 PMC8523415

[nph71415-bib-0050] Feng X , Zheng J , Irisarri I , Yu H , Zheng B , Ali Z , de Vries S , Keller J , Furst‐Jansen JMR , Dadras A *et al*. 2024. Genomes of multicellular algal sisters to land plants illuminate signaling network evolution. Nature Genetics 56: 1018–1031. 38693345 10.1038/s41588-024-01737-3 PMC11096116

[nph71415-bib-0051] Floyd GL , Stewart KD , Mattox KR . 1972. Cellular organization, mitosis, and cytokinesis in the ulotrichalean alga, *Klebsormidium* . Journal of Phycology 8: 176–184.

[nph71415-bib-0052] Foissner I , Wasteneys GO . 2014. Characean internodal cells as a model system for the study of cell organization. International Review of Cell and Molecular Biology 311: 307–364. 24952921 10.1016/B978-0-12-800179-0.00006-4

[nph71415-bib-0053] Fowke LC , Pickett‐Heaps JD . 1969a. Cell Division in *Spirogyra*. I. Mitosis. Journal of Phycology 5: 240–259. 27096344 10.1111/j.1529-8817.1969.tb02609.x

[nph71415-bib-0054] Fowke LC , Pickett‐Heaps JD . 1969b. Cell Division in *Spirogyra*. II. Cytokinesis. Journal of Phycology 5: 273–281. 27096443 10.1111/j.1529-8817.1969.tb02614.x

[nph71415-bib-0055] From the American Association of Neurological Surgeons AANS, ASNR, CIRSE, CIRA, CNS, ESMINT, ESNR, ESO, SCAI, SIR, SNIS, WSO , Sacks D , Baxter B , Campbell BCV , Carpenter JS , Cognard C , Dippel D , Eesa M , Fischer U , Hausegger K *et al*. 2018. Multisociety consensus quality improvement revised consensus statement for endovascular therapy of acute ischemic stroke. International Journal of Stroke 13: 612–632.29786478

[nph71415-bib-0056] Fürst‐Jansen JMR , de Vries S , de Vries J . 2020. Evo‐physio: on stress responses and the earliest land plants. Journal of Experimental Botany 71: 3254–3269. 31922568 10.1093/jxb/eraa007 PMC7289718

[nph71415-bib-0057] Goldbecker ES , de Vries J . 2025. Systems biology of streptophyte cell evolution. Annual Review in Plant Biology 76: 493–522. 10.1146/annurev-arplant-083123-060254 39819561

[nph71415-bib-0058] Goldbecker ES , Irisarri I , de Vries J . 2024. Recurrent evolution of seaweed body plan complexity among photosynthetic eukaryotes. Molecular Plant 17: 999–1001. 38835169 10.1016/j.molp.2024.06.001

[nph71415-bib-0059] Goldbecker ES , Varshney D , Holzhausen A , Darienko T , Zhou H , Dadras A , Pfeifer L , Pröschold T , Lopez‐Gomez E , Woelken E *et al*. 2025. The *Spirogyra* genome: signatures of shared and divergent division and differentiation. *bioRxiv* . doi: 10.1101/2025.10.09.681428.

[nph71415-bib-0060] Gorelova V , Sprakel J , Weijers D . 2021. Plant cell polarity as the nexus of tissue mechanics and morphogenesis. Nature Plants 7: 1548–1559. 34887521 10.1038/s41477-021-01021-w

[nph71415-bib-0061] Gould SJ . 1989. Wonderful life: the burgess shale and the nature of history. New York, NY, USA: W. W. Norton & Company.

[nph71415-bib-0062] Goyal A , Szarzynska B , Fankhauser C . 2013. Phototropism: at the crossroads of light‐signaling pathways. Trends in Plant Science 18: 393–401. 23562459 10.1016/j.tplants.2013.03.002

[nph71415-bib-0063] Graham LE , Cook ME , Busse JS . 2000. The origin of plants: body plan changes contributing to a major evolutionary radiation. Proceedings of the National Academy of Sciences, USA 97: 4535–4540. 10.1073/pnas.97.9.4535 PMC34322 10781058

[nph71415-bib-0064] Grau‐Bove X , Torruella G , Donachie S , Suga H , Leonard G , Richards TA , Ruiz‐Trillo I . 2017. Dynamics of genomic innovation in the unicellular ancestry of animals. eLife 6: e26036. 28726632 10.7554/eLife.26036 PMC5560861

[nph71415-bib-0065] Häder DP , Wenderoth K . 1977. Role of three basic light reactions in photomovement of desmids. Planta 137: 207–214. 24420655 10.1007/BF00388152

[nph71415-bib-0066] Hall JD , McCourt RM , Delwiche CF . 2008. Patterns of cell division in the filamentous Desmidiaceae, close green algal relatives of land plants. American Journal of Botany 95: 643–654. 21632389 10.3732/ajb.2007210

[nph71415-bib-0067] Hamann T . 2015. The plant cell wall integrity maintenance mechanism‐concepts for organization and mode of action. Plant & Cell Physiology 56: 215–223. 25416836 10.1093/pcp/pcu164

[nph71415-bib-0068] Hamant O , Heisler MG , Jonsson H , Krupinski P , Uyttewaal M , Bokov P , Corson F , Sahlin P , Boudaoud A , Meyerowitz EM *et al*. 2008. Developmental patterning by mechanical signals in *Arabidopsis* . Science 322: 1650–1655. 19074340 10.1126/science.1165594

[nph71415-bib-0069] Harris BJ , Clark JW , Schrempf D , Szollosi GJ , Donoghue PCJ , Hetherington AM , Williams TA . 2022. Divergent evolutionary trajectories of bryophytes and tracheophytes from a complex common ancestor of land plants. Nature Ecology & Evolution 6: 1634–1643. 36175544 10.1038/s41559-022-01885-x PMC9630106

[nph71415-bib-0070] Hart JE , Gardner KH . 2021. Lighting the way: Recent insights into the structure and regulation of phototropin blue light receptors. The Journal of Biological Chemistry 296: 100594. 33781746 10.1016/j.jbc.2021.100594 PMC8086140

[nph71415-bib-0071] Haupt W . 1959. Die Chloroplastendrehung bei *Mougeotia*: I. Mitteilung: Über den quantitativen und qualitativen Lichtbedarf der Schwachlichtbewegung. Planta 53: 484–501.

[nph71415-bib-0072] Herburger K , Holzinger A . 2015. Localization and quantification of callose in the streptophyte green algae *Zygnema* and *Klebsormidium*: correlation with desiccation tolerance. Plant & Cell Physiology 56: 2259–2270. 26412780 10.1093/pcp/pcv139 PMC4650865

[nph71415-bib-0073] Herburger K , Ryan LM , Popper ZA , Holzinger A . 2018. Localisation and substrate specificities of transglycanases in charophyte algae relate to development and morphology. Journal of Cell Science 131: jcs203208. 28827406 10.1242/jcs.203208 PMC5722204

[nph71415-bib-0074] Hess S , Williams SK , Busch A , Irisarri I , Delwiche CF , de Vries S , Darienko T , Roger AJ , Archibald JM , Buschmann H *et al*. 2022. A phylogenomically informed five‐order system for the closest relatives of land plants. Current Biology 32: 4473–4482. 36055238 10.1016/j.cub.2022.08.022 PMC9632326

[nph71415-bib-0075] van den Hoek C , Mann DG , Jahns HM . 1995. Algae: an introduction to phycology. Cambridge, UK: Cambridge Univ Press, 623.

[nph71415-bib-0076] Hori S , Sekimoto H , Abe J . 2012. Properties of cell surface carbohydrates in sexual reproduction of the *Closterium peracerosum–strigosum–littorale* complex (Zygnematophyceae, Charophyta). Phycological Research 60: 254–260.

[nph71415-bib-0077] Ibrahim AY , Khaodeuanepheng NP , Amarasekara DL , Correia JJ , Lewis KA , Fitzkee NC , Hough LE , Whitten ST . 2023. Intrinsically disordered regions that drive phase separation form a robustly distinct protein class. The Journal of Biological Chemistry 299: 102801. 36528065 10.1016/j.jbc.2022.102801 PMC9860499

[nph71415-bib-0078] Ikegaya H , Sonobe S , Murakami K , Shimmen T . 2008. Rhizoid differentiation of *Spirogyra* is regulated by substratum. Journal of Plant Research 121: 571–579. 18839271 10.1007/s10265-008-0182-8

[nph71415-bib-0079] Inze D , De Veylder L . 2006. Cell cycle regulation in plant development. Annual Review of Genetics 40: 77–105. 10.1146/annurev.genet.40.110405.090431 17094738

[nph71415-bib-0080] Irisarri I , Darienko T , Pröschold T , Furst‐Jansen JMR , Jamy M , de Vries J . 2021. Unexpected cryptic species among streptophyte algae most distant to land plants. Proceedings of the Biological Sciences 288: 20212168. 34814752 10.1098/rspb.2021.2168 PMC8611356

[nph71415-bib-0081] Jacob F . 1977. Evolution and tinkering. Science 196: 1161–1166. 860134 10.1126/science.860134

[nph71415-bib-0082] Jarvis MC , Briggs SPH , Knox JP . 2003. Intercellular adhesion and cell separation in plants. Plant, Cell & Environment 26: 977–989.

[nph71415-bib-0083] Kadlubowska JZ . 1984. Conjugatophyceae I (Chlorophyta VIII – Zygnematales). In: Ettl H , Gerloff J , Heynig H , Mollenhauer D , eds. Süßwasserflora von Mitteleuropa 16. Stuttgart, Germany: Gustav Fischer, 532 p.

[nph71415-bib-0084] Kaplan DR , Hagemann W . 1991. The relationship of cell and organism in vascular plants. Bioscience 41: 693–703.

[nph71415-bib-0085] Karsten U , Holzinger A . 2014. Green algae in alpine biological soil crust communities: acclimation strategies against ultraviolet radiation and dehydration. Biodiversity and Conservation 23: 1845–1858. 24954980 10.1007/s10531-014-0653-2 PMC4058318

[nph71415-bib-0086] Kawaguchi YW , Tsuchikane Y , Tanaka K , Taji T , Suzuki Y , Toyoda A , Ito M , Watano Y , Nishiyama T , Sekimoto H *et al*. 2023. Extensive copy number variation explains genome size variation in the unicellular zygnematophycean alga, *Closterium peracerosum‐strigosum‐littorale* complex. Genome Biology and Evolution 15: evad115. 37348049 10.1093/gbe/evad115 PMC10407611

[nph71415-bib-0087] Kiermayer O . 1970. Elektronenmikroskopische Untersuchungen zum Problem der Cytomorphogenese von *Micrasterias denticulata* Bréb. Protoplasma 69: 97–132. 10.1007/BF01623967 1265288

[nph71415-bib-0088] Knoll AH . 2011. The multiple origins of complex multicellularity. Annual Review of Earth and Planetary Sciences 39: 217–239.

[nph71415-bib-0089] Kubo T , Kaida S , Abe J , Saito T , Fukuzawa H , Matsuda Y . 2009. The *Chlamydomonas* hatching enzyme, sporangin, is expressed in specific phases of the cell cycle and is localized to the flagella of daughter cells within the sporangial cell wall. Plant & Cell Physiology 50: 572–583. 19179351 10.1093/pcp/pcp016

[nph71415-bib-0090] Kuhn A , Roosjen M , Mutte S , Dubey SM , Carrillo‐Carrasco VP , Boeren S , Monzer A , Koehorst J , Kohchi T , Nishihama R *et al*. 2024. RAF‐like protein kinases mediate a deeply conserved, rapid auxin response. Cell 187: 130–148. 38128538 10.1016/j.cell.2023.11.021 PMC10783624

[nph71415-bib-0091] Kumar M , Michael S , Alvarado‐Valverde J , Zeke A , Lazar T , Glavina J , Nagy‐Kanta E , Donagh JM , Kalman ZE , Pascarelli S *et al*. 2024. ELM‐the Eukaryotic Linear Motif resource‐2024 update. Nucleic Acids Research 52(D1): D442–D455. 37962385 10.1093/nar/gkad1058 PMC10767929

[nph71415-bib-0092] Kumari P , Dahiya P , Livanos P , Zergiebel L , Kolling M , Poeschl Y , Stamm G , Hermann A , Abel S , Muller S *et al*. 2021. IQ67 DOMAIN proteins facilitate preprophase band formation and division‐plane orientation. Nature Plants 7: 739–747. 34031540 10.1038/s41477-021-00923-z

[nph71415-bib-0093] Kunz CF , Abreu IN , Darienko T , Fürst‐Jansen JMR , Feussner K , Feussner I , Lorenz M , de Vries J . 2026. Chemodiverse cell system responses to UV in an algal sister of land plants. Current Biology 36: 2673–2687. 42092354 10.1016/j.cub.2026.04.015

[nph71415-bib-0094] Kunz CF , de Vries S , de Vries J . 2024a. Plant terrestrialization: an environmental pull on the evolution of multi‐sourced streptophyte phenolics. Philosophical Transactions of the Royal Society of London. Series B, Biological Sciences 379: 20230358. 39343031 10.1098/rstb.2023.0358 PMC11528360

[nph71415-bib-0095] Kunz CF , Goldbecker ES , Darienko T , de Vries J . 2024b. Genome evolution: Zygnematophyceae on ice. New Phytologist 244: 1125–1127. 39001590 10.1111/nph.19960

[nph71415-bib-0096] Kunz CF , Goldbecker ES , de Vries J . 2025. Functional genomic perspectives on plant terrestrialization. Trends in Genetics 41: 617–629. 40155238 10.1016/j.tig.2025.02.006

[nph71415-bib-0097] Kurtovic K , Vosolsobe S , Nedved D , Muller K , Dobrev PI , Schmidt V , Piszczek P , Kuhn A , Smoljan A , Fisher TJ *et al*. 2025. The role of indole‐3‐acetic acid and characterization of PIN transporters in complex streptophyte alga *Chara braunii* . New Phytologist 246: 1066–1083. 40047465 10.1111/nph.70019 PMC11982790

[nph71415-bib-0098] Landrein B , Kiss A , Sassi M , Chauvet A , Das P , Cortizo M , Laufs P , Takeda S , Aida M , Traas J *et al*. 2015. Mechanical stress contributes to the expression of the STM homeobox gene in Arabidopsis shoot meristems. eLife 4: e07811. 26623515 10.7554/eLife.07811 PMC4666715

[nph71415-bib-0099] Lewis LA , McCourt RM . 2004. Green algae and the origin of land plants. American Journal of Botany 91: 1535–1556. 21652308 10.3732/ajb.91.10.1535

[nph71415-bib-0100] Li FW , Melkonian M , Rothfels CJ , Villarreal JC , Stevenson DW , Graham SW , Wong GK , Pryer KM , Mathews S . 2015. Phytochrome diversity in green plants and the origin of canonical plant phytochromes. Nature Communications 6: 7852. 10.1038/ncomms8852 PMC4525182 26215968

[nph71415-bib-0101] Lokhorst GM . 1996. Comparative taxonomic studies on the genus *Klebsormidium* (Charophyceae) in Europe. Cryptogamic Studies 5: 1–132.

[nph71415-bib-0102] Lokhorst GM , Sluiman HJ , Star W . 1988. The ultrastructure of mitosis and cytokinesis in the sarcinoid *Chlorokybus atmophyticus* (Chlorophyta, Charophyceae) revealed by rapid freeze fixation and freeze substitution. Journal of Phycology 24: 237–248.

[nph71415-bib-0103] Lokhorst GM , Star W . 1985. Ultrastructure of mitosis and cytokinesis in *Klebsormidium mucosum* nov. comb., formerly *Ulothrix verrucosa* (Chlorophyta). Journal of Phycology 21: 466–476.

[nph71415-bib-0104] Louveaux M , Julien JD , Mirabet V , Boudaoud A , Hamant O . 2016. Cell division plane orientation based on tensile stress in *Arabidopsis thaliana* . Proceedings of the National Academy of Sciences, USA 113: E4294–E4303. 10.1073/pnas.1600677113 PMC4968720 27436908

[nph71415-bib-0105] Luschnig C , Friml J . 2024. Over 25 years of decrypting PIN‐mediated plant development. Nature Communications 15: 9904. 10.1038/s41467-024-54240-y PMC11567971 39548100

[nph71415-bib-0106] Lütz‐Meindl U , Brosch‐Salomon S . 2000. Cell wall secretion in the green alga *Micrasterias* . Journal of Microscopy 198(Pt 3): 208–217. 10849199 10.1046/j.1365-2818.2000.00699.x

[nph71415-bib-0107] Manton I , Ettl H . 1965. Observations on the fine structure of *Mesostigma viride* Lauterborn. Journal of the Linnean Society of London, Botany 59: 175–184.

[nph71415-bib-0108] Marchant HJ , Pickett‐Heaps JD . 1973. Mitosis and cytokinesis in *Coleochaete scutata* . Journal of Phycology 9: 461–471.

[nph71415-bib-0109] Marin B , Melkonian M . 1999. Mesostigmatophyceae, a new class of streptophyte green algae revealed by SSU rRNA sequence comparisons. Protist 150: 399–417. 10714774 10.1016/S1434-4610(99)70041-6

[nph71415-bib-0110] Matsuda Y . 1988. The *Chlamydomonas* cell walls and their degrading enzymes. Japanese Journal of Physiology 36: 246–264.

[nph71415-bib-0111] Matsuda Y , Saito T , Yamaguchi T , Kawase H . 1985. Cell wall lytic enzyme released by mating gametes of *Chlamydomonas reinhardtii* is a metalloprotease and digests the sodium perchlorate‐insoluble. Journal of Biological Chemistry 260: 6373–6377. 3888980

[nph71415-bib-0112] Mayer U , Jürgens G . 2004. Cytokinesis: lines of division taking shape. Current Opinion in Plant Biology 7: 599–604. 15337104 10.1016/j.pbi.2004.07.008

[nph71415-bib-0113] McCourt RM , Lewis LA , Strother PK , Delwiche CF , Wickett NJ , de Vries J , Bowman JL . 2023. Green land: Multiple perspectives on green algal evolution and the earliest land plants. American Journal of Botany 110: e16175. 37247371 10.1002/ajb2.16175

[nph71415-bib-0114] Meindl U . 1993. *Micrasterias* cells as a model system for research on morphogenesis. Microbiological Reviews 57: 415–433. 7687738 10.1128/mr.57.2.415-433.1993 PMC372917

[nph71415-bib-0115] Mikhailyuk T , Lukesova A , Glaser K , Holzinger A , Obwegeser S , Nyporko S , Friedl T , Karsten U . 2018. New taxa of streptophyte algae (Streptophyta) from terrestrial habitats revealed using an integrative approach. Protist 169: 406–431. 29860113 10.1016/j.protis.2018.03.002 PMC6071840

[nph71415-bib-0116] Miller JH , Greany RH . 1974. Determination of rhizoid orientation by light and darkness in germinating spores of *Onoclea sensibilis* . American Journal of Botany 61: 296–302.

[nph71415-bib-0117] Mix M . 1972. Die Feinstruktur der Zellwände bei Mesotaeniaceae und Gonatozygaceae mit einer vergleichenden Betrachtung der verschiedenen Wandtypen der Conjugatophyceae und über deren systematischen Wert. Archiv für Mikrobiologie 81: 197–220.

[nph71415-bib-0118] Mix M . 1973. Die Feinstruktur der Zellwände der Conjugaten und ihre systematische Bedeutung. Beih Nova Hedwigia 42: 179–194.

[nph71415-bib-0119] Morris JL , Puttick MN , Clark JW , Edwards D , Kenrick P , Pressel S , Wellman CH , Yang Z , Schneider H , Donoghue PCJ . 2018. The timescale of early land plant evolution. Proceedings of the National Academy of Sciences, USA 115: E2274–E2283. 10.1073/pnas.1719588115 PMC5877938 29463716

[nph71415-bib-0120] Müller A , Guan C , Galweiler L , Tänzler P , Huijser P , Marchant A , Parry G , Bennett M , Wisman E , Palme K . 1998. AtPIN2 defines a locus of *Arabidopsis* for root gravitropism control. EMBO Journal 17: 6903–6911. 9843496 10.1093/emboj/17.23.6903 PMC1171038

[nph71415-bib-0121] Muller S , Han S , Smith LG . 2006. Two kinesins are involved in the spatial control of cytokinesis in *Arabidopsis thaliana* . Current Biology 16: 888–894. 16682350 10.1016/j.cub.2006.03.034

[nph71415-bib-0122] Mulvey H , Dolan L . 2023. RHO of plant signaling was established early in streptophyte evolution. Current Biology 33: 5515–5525. 38039969 10.1016/j.cub.2023.11.007

[nph71415-bib-0123] Muñoz‐Gomez SA , Bilolikar G , Wideman JG , Geiler‐Samerotte K . 2021. Constructive neutral evolution 20 years later. Journal of Molecular Evolution 89: 172–182. 33604782 10.1007/s00239-021-09996-y PMC7982386

[nph71415-bib-0124] Mutte SK , Kato H , Rothfels C , Melkonian M , Wong GK‐S , Weijers D . 2018. Origin and evolution of the nuclear auxin response system. eLife 7: e33399. 29580381 10.7554/eLife.33399 PMC5873896

[nph71415-bib-0125] Nakagawa Y , Katagiri T , Shinozaki K , Qi Z , Tatsumi H , Furuichi T , Kishigami A , Sokabe M , Kojima I , Sato S *et al*. 2007. *Arabidopsis* plasma membrane protein crucial for Ca2+ influx and touch sensing in roots. Proceedings of the National Academy of Sciences, USA 104: 3639–3644. 10.1073/pnas.0607703104 PMC1802001 17360695

[nph71415-bib-0126] Nelson DR , Mystikou A , Jaiswal A , Rad‐Menendez C , Preston MJ , De Boever F , El Assal DC , Daakour S , Lomas MW , Twizere JC *et al*. 2024. Macroalgal deep genomics illuminate multiple paths to aquatic, photosynthetic multicellularity. Molecular Plant 17: 747–771. 38614077 10.1016/j.molp.2024.03.011

[nph71415-bib-0127] Niklas KJ . 2014. The evolutionary‐developmental origins of multicellularity. American Journal of Botany 101: 6–25. 24363320 10.3732/ajb.1300314

[nph71415-bib-0128] Niklas KJ , Newman SA . 2013. The origins of multicellular organisms. Evolution & Development 15: 41–52. 23331916 10.1111/ede.12013

[nph71415-bib-0129] Niklas KJ , Newman SA . 2020. The many roads to and from multicellularity. Journal of Experimental Botany 71: 3247–3253. 31819969 10.1093/jxb/erz547 PMC7289717

[nph71415-bib-0130] Nishiyama T , Sakayama H , De Vries J , Buschmann H , Saint‐Marcoux D , Ullrich KK , Haas FB , Vanderstraeten L , Becker D , Lang D . 2018. The *Chara* genome: secondary complexity and implications for plant terrestrialization. Cell 174: 448–464. 30007417 10.1016/j.cell.2018.06.033

[nph71415-bib-0131] Ohtaka K , Hori K , Kanno Y , Seo M , Ohta H . 2017. Primitive auxin response without TIR1 and Aux/IAA in the charophyte alga *Klebsormidium nitens* . Plant Physiology 174: 1621–1632. 28533212 10.1104/pp.17.00274 PMC5490900

[nph71415-bib-0132] Ohtaka K , Sekimoto H . 2023. Zygnematophycean algae: possible models for cellular and evolutionary biology. Seminars in Cell and Developmental Biology 134: 59–68. 35430142 10.1016/j.semcdb.2022.03.042

[nph71415-bib-0133] One Thousand Plant Transcriptomes I . 2019. One thousand plant transcriptomes and the phylogenomics of green plants. Nature 574: 679–685. 31645766 10.1038/s41586-019-1693-2 PMC6872490

[nph71415-bib-0134] Ozawa Y , Anbo H , Ota M , Fukuchi S . 2023. Classification of proteins inducing liquid‐liquid phase separation: sequential, structural and functional characterization. Journal of Biochemistry 173: 255–264. 36575582 10.1093/jb/mvac106 PMC10064836

[nph71415-bib-0135] Palla E . 1894. Über eine neue, pyrenoidlose Art und Gattung der Conjugaten. Berichte der Deutschen Botanischen Gesellschaft 12: 228–236.

[nph71415-bib-0136] Pelletier S , Van Orden J , Wolf S , Vissenberg K , Delacourt J , Ndong YA , Pelloux J , Bischoff V , Urbain A , Mouille G *et al*. 2010. A role for pectin de‐methylesterification in a developmentally regulated growth acceleration in dark‐grown Arabidopsis hypocotyls. New Phytologist 188: 726–739. 20819179 10.1111/j.1469-8137.2010.03409.x

[nph71415-bib-0137] Pichrtová M , Holzinger A , Kulichová J , Ryšánek D , Šoljaková T , Trumhová K , Nemcova Y . 2018. Molecular and morphological diversity of *Zygnema* and *Zygnemopsis* (Zygnematophyceae, Streptophyta) from Svalbard (high Arctic). European Journal of Phycology 53: 492–508. 30487730 10.1080/09670262.2018.1476920 PMC6235541

[nph71415-bib-0138] Pickett‐Heaps JD . 1975. Green algae: Structure, reproduction and evolution in selected genera. Sunderland, MA: Sinauer Associates, 606 p.

[nph71415-bib-0139] Pickett‐Heaps JD , Fowke LC . 1970. Mitosis, cytokinesis, and cell elongation in the desmid, *Closterium littorale* . Journal of Phycology 6: 189–215.

[nph71415-bib-0140] Pickett‐Heaps JD , Fowke LC . 1971. Conjugation in the desmid *Closterium littorale* . Journal of Phycology 7: 37–50.

[nph71415-bib-0141] Pillitteri LJ , Guo X , Dong J . 2016. Asymmetric cell division in plants: mechanisms of symmetry breaking and cell fate determination. Cellular and Molecular Life Sciences 73: 4213–4229. 27286799 10.1007/s00018-016-2290-2 PMC5522748

[nph71415-bib-0142] Prieto G , Rodriguez JA , Fullaondo A . 2024. Enhancing prediction of short linear protein motifs with Wregex 3.0. Computational and Structural Biotechnology Journal 23: 2978–2984. 39135888 10.1016/j.csbj.2024.07.013 PMC11318550

[nph71415-bib-0143] Pringsheim M . 1862. XXXIII.—On the pro‐embryos of the Charae. Annals and Magazine of Natural History 10: 321–326.

[nph71415-bib-0144] Printz H . 1964. Die Chaetophoralen der Binnengewässer. Hydrobiologia 24: 1–376.

[nph71415-bib-0145] Procházková L , Mojzes P , Racek J , Nedbalova L , Remias D . 2025. Phenolic iron complexes protect glacier ice algae (Zygnematophyceae) against excessive UV and VIS irradiation. Environmental Microbiology Reports 17: e70149. 40654230 10.1111/1758-2229.70149 PMC12257149

[nph71415-bib-0146] Prochnik SE , Umen J , Nedelcu AM , Hallmann A , Miller SM , Nishii I , Ferris P , Kuo A , Mitros T , Fritz‐Laylin LK . 2010. Genomic analysis of organismal complexity in the multicellular green alga *Volvox carteri* . Science 329: 223–226. 20616280 10.1126/science.1188800 PMC2993248

[nph71415-bib-0147] Puttick MN , Morris JL , Williams TA , Cox CJ , Edwards D , Kenrick P , Pressel S , Wellman CH , Schneider H , Pisani D *et al*. 2018. The interrelationships of land plants and the nature of the ancestral embryophyte. Current Biology 28: 733–745. 29456145 10.1016/j.cub.2018.01.063

[nph71415-bib-0148] Remias D , Holzinger A , Aigner S , Lütz C . 2012a. Ecophysiology and ultrastructure of *Ancylonema nordenskiöldii* (Zygnematales, Streptophyta), causing brown ice on glaciers in Svalbard (high arctic). Polar Biology 35: 899–908.

[nph71415-bib-0149] Remias D , Procházková L . 2023. The first cultivation of the glacier ice alga Ancylonema alaskanum (Zygnematophyceae, Streptophyta): differences in morphology and photophysiology of field vs laboratory strain cells. Journal of Glaciology 69: 1080–1084.

[nph71415-bib-0150] Remias D , Schwaiger S , Aigner S , Leya T , Stuppner H , Lütz C . 2012b. Characterization of an UV‐ and VIS‐absorbing, purpurogallin‐derived secondary pigment new to algae and highly abundant in *Mesotaenium berggrenii* (Zygnematophyceae, Chlorophyta), an extremophyte living on glaciers. FEMS Microbiology Ecology 79: 638–648. 22092588 10.1111/j.1574-6941.2011.01245.x

[nph71415-bib-0151] Renner SS , Sokoloff DD . 2024. The sexual lability hypothesis for the origin of the land plant generation cycle. Current Biology 34: R697–R707. 39043145 10.1016/j.cub.2024.05.029

[nph71415-bib-0152] Rieseberg TP , Dadras A , Darienko T , Post S , Herrfurth C , Fürst‐Jansen JMR , Hohnhorst N , Petroll R , Rensing SA , Pröschold T *et al*. 2025. Time‐resolved oxidative signal convergence across the algae‐embryophyte divide. Nature Communications 16: 1780. 10.1038/s41467-025-56939-y PMC11840003 39971942

[nph71415-bib-0153] Rippin M , Pichrtová M , Arc E , Kranner I , Becker B , Holzinger A . 2019. Metatranscriptomic and metabolite profiling reveals vertical heterogeneity within a *Zygnema* green algal mat from Svalbard (High Arctic). Environmental Microbiology 21: 4283–4299. 31454446 10.1111/1462-2920.14788 PMC6899726

[nph71415-bib-0154] Roberts JA , Elliott KA , Gonzalez‐Carranza ZH . 2002. Abscission, dehiscence, and other cell separation processes. Annual Review of Plant Biology 53: 131–158. 10.1146/annurev.arplant.53.092701.180236 12221970

[nph71415-bib-0155] Roetzer J , Slovak R , Wallner E‐S , Edelbacher N , Asper B , Deiber S , Seitner S , Colombini M , Dolan L . 2026. PHOTOTROPIN‐mediated blue light signaling orients the asymmetry of *Marchantia polymorpha* spores. *bioRxiv*. doi: 10.64898/2026.03.22.713455.

[nph71415-bib-1002] Rogers CE , Domozych DS , Stewart KD , Mattox KR . 1981. The flagellar apparatus of *Mesostigma viride* (Prasinophyceae): multilayered structures in a scaly green flagellate. Plant Systematics and Evolution 138: 247–258.

[nph71415-bib-0156] Rogers CE , Mattox KR , Stewart KD . 1980. The zoospore of *Chlorokybus atmophyticus*, a charophyte with sarcinoid growth habit. American Journal of Botany 67: 774–783.

[nph71415-bib-0157] Sawitzky H , Grolig F . 1995. Phragmoplast of the green alga *Spirogyra* is functionally distinct from the higher plant phragmoplast. The Journal of Cell Biology 130: 1359–1371. 7559758 10.1083/jcb.130.6.1359 PMC2120580

[nph71415-bib-0158] Schleiden MJ . 1838. Beiträge zur Phytogenesis. Archiv für Anatomie, Physiologie und Wissenschaftliche Medicin 1838: 137–176.

[nph71415-bib-0159] Schlösser UG . 1976. Entwicklungsstadien‐ und sippenspezifische Zellwand‐Autolysine bei der Freisetzung von Fortpflanzungszellen in der Gattung *Chlamydomonas* . Berichte der Deutschen Botanischen Gesellschaft 89: 1–56.

[nph71415-bib-0160] Schlösser UG . 1981. Algal‐degrading enzymes – Autolysines. In: Tanner W , Loewus FA , eds. Plant carbohydrates II. Encyclopedia of plant physiology, new series 13B. Berlin: Springer, 333–351.

[nph71415-bib-0161] Schlösser UG . 1984. Species‐specific sporangium autolysins (cell‐wall‐dissolving enzymes) in the genus *Chlamydomonas* . In: Irvine DEG , John DM , eds. *Systematics of the green algae*. Systematics Association Special, vol. 27. London, UK: Academic Press, 409–418.

[nph71415-bib-0162] Schlösser UG . 1987. Action of cell wall autolysins in asexual reproduction of filamentous green algae: Evidence and species specificity. In: Wiessner W , Robinson DG , Starr RC , eds. Algal development (Molecular and cellular aspects). Berlin Heidelberg: Springer, 75–82.

[nph71415-bib-1003] Schlösser UG , Renkert U . 1992. Growth and Fragmentation of *Zygnema circumcarinatum*. Göttingen: IWF C1770. available under https://av.tib.eu/media/14346.

[nph71415-bib-0163] Schmit AC , Nick P . 2008. Microtubules and the evolution of mitosis. Plant Cell Monographs 11: 233–266.

[nph71415-bib-0164] Schwann T . 1839. Mikroskopische Untersuchungen über die Uebereinstimmung in der Struktur und dem Wachsthum der Thiere und Pflanzen. Berlin: Sander. 21516882

[nph71415-bib-0165] Sekimoto H , Abe J , Tsuchikane Y . 2012. New insights into the regulation of sexual reproduction in *Closterium* . International Review of Cell and Molecular Biology 297: 309–338. 22608564 10.1016/B978-0-12-394308-8.00014-5

[nph71415-bib-1001] Skokan R , Medvecka E , Viaene T , Vosolsobe S , Zwiewka M , Muller K , Skupa P , Karady M , Zhang Y , Janacek DP *et al*. 2019. PIN‐driven auxin transport emerged early in streptophyte evolution. Nature Plants 5(11): 1114–1119. 31712756 10.1038/s41477-019-0542-5

[nph71415-bib-0166] Spinner L , Gadeyne A , Belcram K , Goussot M , Moison M , Duroc Y , Eeckhout D , De Winne N , Schaefer E , Van De Slijke E *et al*. 2013. A protein phosphatase 2A complex spatially controls plant cell division. Nature Communications 4: 1863. 10.1038/ncomms2831 23673648

[nph71415-bib-0167] Stancheva R , Hall JD , Herburger K , Lewis LA , McCourt RM , Sheath RG , Holzinger A . 2014. Phylogenetic position of *Zygogonium ericetorum* (Zygnematophyceae, Charophyta) from a high alpine habitat and ultrastructural characterization of unusual aplanospores. Journal of Phycology 50: 790–803. 25810560 10.1111/jpy.12229 PMC4370237

[nph71415-bib-0168] Strassert JFH , Irisarri I , Williams TA , Burki F . 2021. A molecular timescale for eukaryote evolution with implications for the origin of red algal‐derived plastids. Nature Communications 12: 1879. 10.1038/s41467-021-22044-z PMC7994803 33767194

[nph71415-bib-0169] Suetsugu N , Mittmann F , Wagner G , Hughes J , Wada M . 2005. A chimeric photoreceptor gene, NEOCHROME, has arisen twice during plant evolution. Proceedings of the National Academy of Sciences, USA 102: 13705–13709. 10.1073/pnas.0504734102 PMC1224637 16174755

[nph71415-bib-0170] Suo J , Chen S , Zhao Q , Shi L , Dai S . 2015. Fern spore germination in response to environmental factors. Frontiers in Biology 10: 358–376.

[nph71415-bib-0171] Tee EE , Faulkner C . 2024. Plasmodesmata and intercellular molecular traffic control. New Phytologist 243: 32–47. 38494438 10.1111/nph.19666

[nph71415-bib-0172] Tsuchikane Y , Fujii T , Ito M , Sekimoto H . 2005. A sex pheromone, protoplast release‐inducing protein (PR‐IP) inducer, induces sexual cell division and production of PR‐IP in *Closterium* . Plant & Cell Physiology 46: 1472–1476. 15970600 10.1093/pcp/pci158

[nph71415-bib-0173] Tsuchikane Y , Kokubun Y , Sekimoto H . 2010. Characterization and molecular cloning of conjugation‐regulating sex pheromones in homothallic *Closterium* . Plant & Cell Physiology 51: 1515–1523. 20656896 10.1093/pcp/pcq110

[nph71415-bib-0174] Tsuchikane Y , Sekimoto H . 2019. The genus *Closterium*, a new model organism to study sexual reproduction in streptophytes. New Phytologist 221: 99–104. 29992575 10.1111/nph.15334

[nph71415-bib-0175] Tsuchikane Y , Watanabe M , Kawaguchi YW , Uehara K , Nishiyama T , Sekimoto H , Tsuchimatsu T . 2024. Diversity of genome size and chromosome number in homothallic and heterothallic strains of the *Closterium peracerosum‐strigosum‐littorale* complex (Desmidiales, Zygnematophyceae, Streptophyta). Journal of Phycology 60: 654–667. 38678594 10.1111/jpy.13457

[nph71415-bib-0176] Umen J , Herron MD . 2021. Green algal models for multicellularity. Annual Review of Genetics 55: 603–632. 10.1146/annurev-genet-032321-091533 34546795

[nph71415-bib-0177] Umen JG . 2014. Green algae and the origins of multicellularity in the plant kingdom. Cold Spring Harbor Perspectives in Biology 6: a016170. 25324214 10.1101/cshperspect.a016170 PMC4413236

[nph71415-bib-0178] Vaahtera L , Schulz J , Hamann T . 2019. Cell wall integrity maintenance during plant development and interaction with the environment. Nature Plants 5: 924–932. 31506641 10.1038/s41477-019-0502-0

[nph71415-bib-0179] Vanneste S , Pei Y , Friml J . 2025. Mechanisms of auxin action in plant growth and development. Nature Reviews. Molecular Cell Biology 26: 648–666. 40389696 10.1038/s41580-025-00851-2

[nph71415-bib-0180] Vogelmann TC , Bassel AR , Miller JH . 1981. Effects of microtubule‐inhibitors on nuclear migration and rhizoid differentiation in germinating fern spores (*Onoclea sensibilis*). Protoplasma 109: 295–316.

[nph71415-bib-0181] de Vries J , Archibald JM . 2018. Plant evolution: landmarks on the path to terrestrial life. New Phytologist 217: 1428–1434. 29318635 10.1111/nph.14975

[nph71415-bib-0182] Walker KL , Muller S , Moss D , Ehrhardt DW , Smith LG . 2007. *Arabidopsis* TANGLED identifies the division plane throughout mitosis and cytokinesis. Current Biology 17: 1827–1836. 17964159 10.1016/j.cub.2007.09.063 PMC2134921

[nph71415-bib-0183] Wegner L , Porth ML , Ehlers K . 2023. Multicellularity and the need for communication‐A systematic overview on (algal) plasmodesmata and other types of symplasmic cell connections. Plants 12: 3342. 37765506 10.3390/plants12183342 PMC10536634

[nph71415-bib-0184] Wickett NJ , Mirarab S , Nguyen N , Warnow T , Carpenter E , Matasci N , Ayyampalayam S , Barker MS , Burleigh JG , Gitzendanner MA *et al*. 2014. Phylotranscriptomic analysis of the origin and early diversification of land plants. Proceedings of the National Academy of Sciences, USA 111: E4859–E4868. 10.1073/pnas.1323926111 PMC4234587 25355905

[nph71415-bib-0185] Wodniok S , Brinkmann H , Glockner G , Heidel AJ , Philippe H , Melkonian M , Becker B . 2011. Origin of land plants: do conjugating green algae hold the key? BMC Evolutionary Biology 11: 104. 21501468 10.1186/1471-2148-11-104 PMC3088898

[nph71415-bib-0186] Wood NL , Berliner MD . 1979. Effects of indoleacetic acid on the desmid *Micrasterias thomasiana* . Plant Science Letters 16: 285–289.

[nph71415-bib-0187] Woudenberg S , Plackett ARG , Hao Z , Suzuki H , Baez LA , Borassi C , Hamann T , Ueda M , Langdale JA , Sprakel J *et al*. 2025. Transgenerational polarity axis inheritance during *Ceratopteris* embryogenesis. *bioRxiv* . doi: 10.1101/2025.08.29.673061.

[nph71415-bib-0188] Zarsky J , Zarsky V , Hanacek M , Zarsky V . 2021. Cryogenian glacial habitats as a plant terrestrialisation cradle ‐ The origin of the anydrophytes and Zygnematophyceae Split. Frontiers in Plant Science 12: 735020. 35154170 10.3389/fpls.2021.735020 PMC8829067

[nph71415-bib-0189] Zegers JMS , Garcia Ramirez GX , Harzen A , Stolze SC , Salem M , Ziplys A , Mora‐Ramírez I , Shpilman M , Mosquna A , Schmitt K *et al*. 2026b. Homologous ABA‐independent kinase tracks coalesced into osmotic stress circuits during plant terrestrialization. *bioRxiv* . doi: 10.64898/2026.05.21.726866.

[nph71415-bib-0190] Zegers JMS , Irisarri I , de Vries S , de Vries J . 2024. Evolving circuitries in plant signaling cascades. Journal of Cell Science 137: jcs261712. 39239891 10.1242/jcs.261712

[nph71415-bib-0191] Zegers JMS , Pfeifer L , Darienko T , Schmitt K , Dorsch CA , Kunz CF , Braus GH , Abreu IN , Feussner I , Classen B *et al*. 2026a. Systems acclimation to osmotic stress in zygnematophyte cells. Nature Communications 17: 755. 10.1038/s41467-026-68329-z PMC12820192 41545391

